# Targeting a xenobiotic transporter to ameliorate vincristine-induced sensory neuropathy

**DOI:** 10.1172/jci.insight.164646

**Published:** 2023-07-24

**Authors:** Yang Li, Thomas Drabison, Mahesh Nepal, Richard H. Ho, Alix F. Leblanc, Alice A. Gibson, Yan Jin, Wenjian Yang, Kevin M. Huang, Muhammad Erfan Uddin, Mingqing Chen, Duncan F. DiGiacomo, Xihui Chen, Sobia Razzaq, Jeffrey R. Tonniges, Dana M. McTigue, Alice S. Mims, Maryam B. Lustberg, Yijia Wang, Amanda B. Hummon, William E. Evans, Sharyn D. Baker, Guido Cavaletti, Alex Sparreboom, Shuiying Hu

**Affiliations:** 1 Division of Pharmaceutics and Pharmacology, College of Pharmacy & Comprehensive Cancer Center, and; 2 Division of Outcomes and Translational Sciences, College of Pharmacy & Comprehensive Cancer Center, The Ohio State University, Columbus, Ohio, USA.; 3 Vanderbilt University Medical Center, Nashville, Tennessee, USA.; 4 Department of Pharmaceutical Sciences, St. Jude Children’s Research Hospital, Memphis, Tennessee, USA.; 5 Campus Microscopy and Imaging Facility,; 6 The Belford Center for Spinal Cord Injury & Department of Neuroscience, College of Medicine, and; 7 Division of Hematology, Department of Internal Medicine, The Ohio State University, Columbus, Ohio, USA.; 8 The Breast Center at Smilow Cancer Hospital at Yale, New Haven, Connecticut, USA.; 9 Department of Chemistry and Biochemistry & Comprehensive Cancer Center, The Ohio State University, Columbus, Ohio, USA.; 10 Experimental Neurology Unit and Milan Center for Neuroscience, School of Medicine and Surgery, University of Milano-Bicocca, Monza, Italy.

**Keywords:** Oncology, Pharmacology, Toxins/drugs/xenobiotics, Transport

## Abstract

Vincristine is a widely used chemotherapeutic drug for the treatment of multiple malignant diseases that causes a dose-limiting peripheral neurotoxicity. There is no clinically effective preventative treatment for vincristine-induced sensory peripheral neurotoxicity (VIPN), and mechanistic details of this side effect remain poorly understood. We hypothesized that VIPN is dependent on transporter-mediated vincristine accumulation in dorsal root ganglion neurons. Using a xenobiotic transporter screen, we identified OATP1B3 as a neuronal transporter regulating the uptake of vincristine. In addition, genetic or pharmacological inhibition of the murine orthologue transporter OATP1B2 protected mice from various hallmarks of VIPN — including mechanical allodynia, thermal hyperalgesia, and changes in digital maximal action potential amplitudes and neuronal morphology — without negatively affecting plasma levels or antitumor effects of vincristine. Finally, we identified α-tocopherol from an untargeted metabolomics analysis as a circulating endogenous biomarker of neuronal OATP1B2 function, and it could serve as a companion diagnostic to guide dose selection of OATP1B-type transport modulators given in combination with vincristine to prevent VIPN. Collectively, our findings shed light on the fundamental basis of VIPN and provide a rationale for the clinical development of transporter inhibitors to prevent this debilitating side effect.

## Graphical abstract

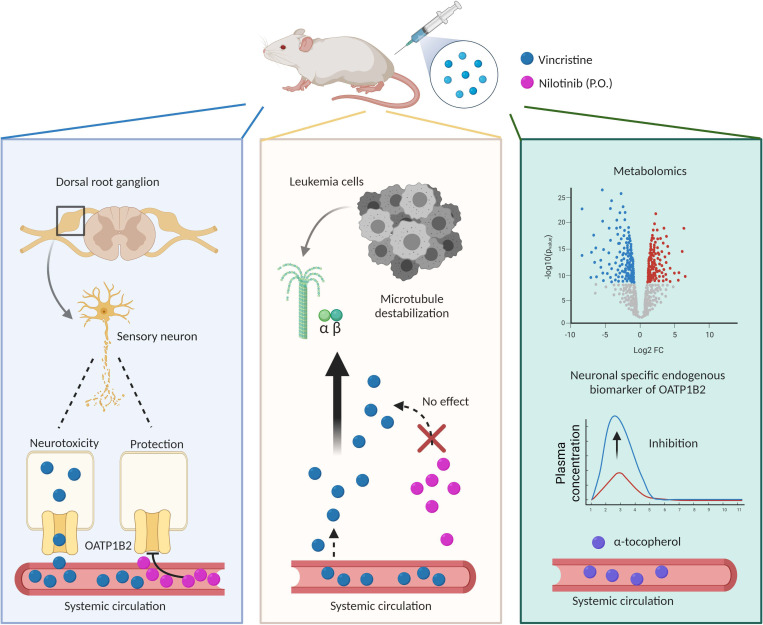

## Introduction

Many tubulin poisons used in the chemotherapeutic treatment of cancer induce a chronic, dose-dependent sensory peripheral neurotoxicity that is characterized by tingling, numbness, increased sensitivity to cold and touch, and burning pain of the distal extremities. The incidence of this side effect is particularly high in the case of vincristine and can occur in up to 100% of patients with cancer, depending on vincristine doses, the number of treatment cycles, and the methods used to obtain neurotoxicity information (1). With continued dosing of vincristine, the painful symptoms increase in severity and can persist for years or even cause a lifelong functional impairment that impacts quality of life (2). The mechanistic basis of vincristine-induced sensory peripheral neurotoxicity (VIPN) remains uncertain (3), although prior studies have demonstrated that the agent induces injury to sensory neurons, morphological and biochemical alterations in dorsal root ganglion (DRG) neurons, hyperplasia/hypertrophy of macrophages in the peripheral nervous system, neuroinflammatory mechanisms involving release of interleukin-1β from macrophages, and increases in microglial and astrocyte activation within the spinal cord (4–7).

Over the last 4 decades, various approaches have been proposed to predict, prevent, and/or treat VIPN (8, 9). The predictive strategies have predominantly focused on the search for hereditary biomarkers that could identify patients at increased risk of toxicity through candidate gene or genome-wide association studies (10). However, the findings from studies done to date have often identified nonoverlapping single or pathway biomarker associations that preclude immediate clinical implementation. In addition, the decision to act on a toxicity biomarker is hampered in many diseases by the lack of available alternative treatments to replace vincristine and/or the need for a patient-tailored reduction in the vincristine dose to prevent toxicity, which negatively affects disease management (1). Given the increasing numbers of long-term cancer survivors who have received legacy therapy with vincristine (11), the development of new preventative strategies that effectively afford protection against VIPN is urgently needed. Although prophylactic interventions for vincristine have been proposed (12–16), most of these have not been evaluated in humans; thus, their clinical effectiveness remains unclear.

Previous studies have suggested that the severity of neurotoxic effects associated with drugs such as vincristine is directly related to the levels at which they accumulate in DRG neurons within the peripheral nervous system (17). Although the identities of enzymes and efflux transporters involved in the pharmacokinetic profile of vincristine have been reasonably well documented and shown to involve selective metabolism by the cytochrome P450 isoform CYP3A5 (18–20) and transport by several ATP-binding cassette transporters (21), the transport mechanism by which vincristine is taken up into neuronal cells remains unclear (22, 23). In this context, it is noteworthy that many other neurotoxic tubulin poisons, including paclitaxel and ixabepilone, accumulate extensively in DRG neurons (17, 24) — and to a lesser degree in the sciatic nerve and spinal cord (25) — and that the cellular uptake of other microtubule-targeting agents (MTAs) occurs via facilitated mechanisms by specific transporters (26). The involvement of a similar mechanism for vincristine is consistent with the existence of an unidentified uptake transporter of vincristine in various mammalian cells that is saturable, temperature dependent, and sensitive to pharmacological inhibition (27). We hypothesized that identification of the transporter-mediated mechanism of vincristine uptake in DRG neurons may shed light on the etiology of VIPN and may lead to the development of novel therapeutic interventions. Here, we report that the uptake of vincristine in DRG neurons is mediated by the organic anion transporting polypeptides OATP1B2 (mouse) and OATP1B3 (human), which are transporters that can be targeted pharmacologically with a biomarker-driven approach in order to afford neuroprotection without negatively influencing the antitumor properties of vincristine.

## Results

### Identification of vincristine uptake transporters in DRG.

**Figure 1:**
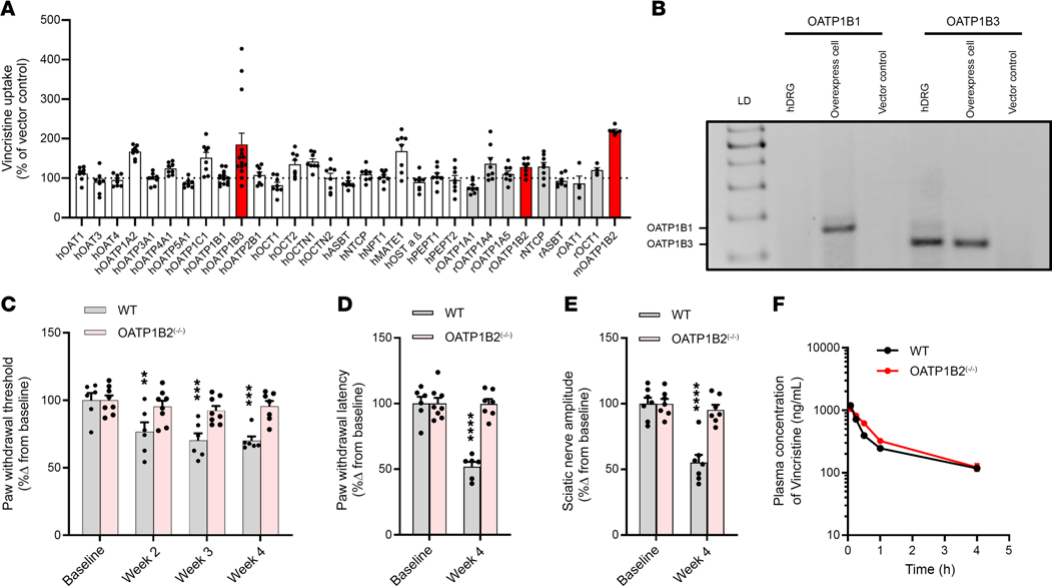
OATP1B2 deficiency attenuates vincristine-induced sensory peripheral neurotoxicity (VIPN). (**A**) Intracellular accumulation of vincristine into HeLa cells overexpressing mouse (m), rat (r), or human (h) transporters, except for mOATP1B2, whose model was generated in HEK293 cells. Relative uptake is expressed as percentage change compared with empty vector controls (*n* = 4–14 per group). (**B**) Expression of the OATP1B1 and OATP1B3 genes in human whole DRG samples (pooled RNA from 21 male/female subjects), by reverse transcription PCR (RT-PCR) (depicted by the 154 bp product for OATP1B1 and 111 bp product for OATP1B3). Human OATP1B1 and OATP1B3 transporter–overexpressed cells were used as positive controls; empty vector controls were used as the negative control. LD, 100 bp ladder. (**C**–**E**) VIPN in WT mice or OATP1B2 deficient mice (OATP1B2^–/–^) at baseline and at 2, 3, and 4 weeks following weekly administrations of vincristine at a dose of 1 mg/kg (cumulative dose 4 mg/kg). Mechanical allodynia (**C**), thermal hyperalgesia (**D**), and sciatic nerve maximal action potential amplitudes (**E**) are expressed as percentage change relative to baseline values (*n* = 6–8 per group). Statistical analysis was performed using a 2-way ANOVA with Bonferroni’s post hoc test. **P < 0.01, ***P < 0.001, ****P < 0.0001, compared with baseline values. (**F**) Plasma concentration-time profile of vincristine (1 mg/kg) in WT mice or OATP1B2^–/–^ mice (*n* = 4 per group). Data are shown as mean ± SEM.

To identify a neuronal uptake transporter of vincristine, we initially performed a screen in HeLa cells engineered to overexpress family members of the class of organic anion transporters (OAT), organic anion transporting polypeptides (OATP), organic cation transporters (OCT), and bile acid/peptide transporters that are known to be involved in the transport of xenobiotics without consideration of their expression levels in DRG neurons. The results of this screen indicate that vincristine was most efficiently transported by OATP1B3, and transport was verified in models overexpressing the orthologous transporter OATP1B2 in both the rat (HeLa cells) and mouse (HEK293 cells) (Figure 1A). Beyond OATP1B-type transporters, vincristine uptake in the initial screen was also increased in HeLa cells overexpressing OATP1A2, OATP1C1, and MATE1, although the 2 former transporters are expressed in the central nervous system rather than in DRG neurons (28, 29) and the latter mediates xenobiotic efflux in DRG neurons, as opposed to uptake (30). In addition, secondary validation studies in HEK293 cell–based models for OATP1A2, OATP1C1, mouse MATE1, and human MATE1 verified that these transporters do not appreciably contribute to the cellular uptake of vincristine or, in the case of MATE1, to VIPN in mice (Supplemental Figure 1, A–E; supplemental material available online with this article; https://doi.org/10.1172/jci.insight.164646DS1). Based on these results, we focused on OATP1B3 as a contributor to vincristine transport in subsequent studies.

Several known protein variants of OATP1B3 associated with single-nucleotide substitutions showed an impaired ability to transport vincristine (Supplemental Figure 2A), as observed previously with other substrates (31), further supporting the notion that vincristine is a transported substrate of OATP1B3. Because of their common allele frequency and evidence for functional relevance in vitro and in vivo, we evaluated the OATP1B3 nucleotide variants 334T>G and 699G>A both singly and in combination and found that the latter rescued OATP1B3 activity (Supplemental Figure 2A). This is consistent with the current understanding (32) that the functional relevance of this double variant is substrate dependent such that, for substrates such as CCK8 (a derivative of the gastrointestinal peptide hormone cholecystokinin) or mycophenolate (an immunosuppressive agent), the combination may lead to significant loss of function, whereas for substrates such as rosuvastatin (a lipid-lowering agent), the double variant rescues function. Although we did not further assess structure-function relationships of the 334T>G or 699G>A variants, it should be pointed out that substrate-dependent transport due to inherited variation has been noted previously in the setting of other xenobiotic transporters, such as OATP1B1 and the sodium taurocholate cotransporting polypeptide NTCP (SLC10A1) (32).

Analogous to our current findings with vincristine, we previously reported that neurotoxic taxanes such as paclitaxel and docetaxel are also transported substrates of OATP1B3 (26, 33–35) as well as of OATP1B2 in mice (36) and rats (36, 37). These findings have been independently verified (38–42) and are consistent with in vitro studies that have demonstrated that vincristine can inhibit OATP1B3 in vitro (31, 39, 43, 44), a common feature of substrates, and that vincristine is itself a transported substrate in OATP1B3-expressing CHO cells (23). These findings support the thesis that uptake carriers capable of transporting vincristine need to be expressed in neuronal tissues such that the drug can be taken up and exert cellular injury.

Although OATP1B-type transporters were originally believed to be liver specific (45), recent studies have indicated that the OATP1B2 gene and protein are detectable in bulk mouse DRG tissue (24), and we observed here that transcripts of OATP1B3, but not of the related transporter OATP1B1, are detectable in whole human DRG samples at levels similar to those observed in our engineered HEK293 cells (Figure 1B). OATP1B1 was considered in this context because it forms a functional unit with OATP1B3 in the human liver and because, in that organ, these 2 proteins together fulfill the same function as the single transporter OATP1B2 in rodents. Recent gene expression analyses have indicated that, within bulk DRG samples, OATP1B2 is preferentially expressed in satellite glial cells (SGCs) (46), which is in accordance with the documented contribution of SGCs to chemotherapy-induced pain phenotypes (47). Nonetheless, further studies are required in pure neuronal populations to directly confirm the expression of OATP1B2 and OATP1B3 in sensory neurons of mice and humans, respectively, and to identify the specific subset of cells that retain transport function.

### Contribution of OATP1B2 to VIPN.

To determine the role of OATP1B2 in VIPN in vivo, we performed comparative studies in WT mice and OATP1B2-deficient (OATP1B2^–/–^) mice (48) receiving 4 weekly 1 mg/kg doses of vincristine (Supplemental Figure 2, B and C). We initially determined the role of OATP1B2 in VIPN with a von Frey test to assess mechanical allodynia, a method that has been extensively used previously to detect VIPN in mice (49–51). Our results using this test suggest that WT mice and OATP1B2^–/–^ mice do not show intrinsic differences in sensitivity to mechanical stimuli at baseline (Figure 1C). However, WT mice experience significantly increased sensitivity to mechanical stimulation after administration of vincristine, where the force to induce a response decreased by about 40% during the course of 4 weekly treatments, while OATP1B2^–/–^ mice exposed to vincristine showed no significant change in sensitivity to mechanical allodynia at any time point (Figure 1C and Supplemental Figure 2, D and E), regardless of sex (Supplemental Figure 2, F and G). These results suggest that OATP1B2^–/–^ mice are fully protected from VIPN similar to observations in animals receiving only the control vehicle. Interestingly, the extent of the decrease in paw withdrawal between days 1 and 3 following a single dose of vincristine is similar to that observed between days 9 and 23, with vincristine administered once every week. Although the basis for this observation is unclear, it suggests that the paw withdrawal threshold is not changing progressively over time as a neuropathy would be expected to develop.

In ensuing experiments, we found that OATP1B2^–/–^ mice were also protected against various other hallmarks of VIPN, including thermal hyperalgesia (Figure 1D) and changes in digital nerve maximal action potential amplitude (AMP) (Figure 1E). To compensate for the lack of a neuron-specific KO model for OATP1B2, we also generated VIPN data in mice with a global knockout of all OATP1A and OATP1B isoforms (OATP1A/1B^–/–^ mice) with or without hepatic-specific expression of human OATP1B3. The results indicate that both the OATP1A/1B^–/–^ mice and the animals with transgenic expression of OATP1B3 in the liver are protected against markers of VIPN (Supplemental Figure 1F). Since OATP1B3 is only known to be expressed in hepatocytes and DRG neurons, this finding provides indirect support for the thesis that the effects observed in the OATP1B2^–/–^ mice are due to reduced uptake of vincristine into the peripheral nervous system.

Since patients treated with vincristine can develop both sensory and motor disturbances such as altered gait and foot drop syndrome (2), we also addressed treatment effects on motor neurons using automated ladder and open field tests and found that, under conditions that cause measurable sensory effects, vincristine did not affect gross motor performance either in WT mice or OATP1B2^–/–^ mice (Supplemental Figure 3). Although gait abnormalities have been documented previously in mice treated with alternate regimens of vincristine (7, 52), these phenotypes were not further evaluated in our subsequent studies, since OATP1B2 is undetectable in murine motor neurons (53).

### Association of OATP1B2 with vincristine pharmacokinetics.

The systemic exposure to vincristine, expressed as area under the plasma concentration-time curve (AUC), following administration of a clinically relevant dose of vincristine (1 mg/kg; i.p.) was unchanged in OATP1B2^–/–^ mice (Figure 1F and Supplemental Table 1), and the observed terminal half-lives of vincristine were not dependent on mouse genotype. Furthermore, we found that the neuronal expression of OATP1A4, the only other vincristine transporter (Figure 1A) with detectable levels in mouse DRG samples (46), was unchanged in OATP1B2^–/–^ mice (Supplemental Figure 4A). This observation is relevant in consideration of the notion that deletion of one transporter can occasionally result in the compensatory overexpression of functionally redundant transporters. However, in this case, OATP1A4 expression levels were not influenced by genetic loss of OATP1B2, and this observation substantiates the validity of the chosen OATP1B2^–/–^ model for our in vivo studies. This is further supported by the finding that the additional deficiency of all OATP1A-type transporters in OATP1A/1B^–/–^ mice had no influence on the vincristine AUC (Supplemental Figure 4B). This suggests that potentially genotype-dependent differences in systemic exposure do not directly affect VIPN and that transporters other than OATP1B2 are unlikely to contribute independently to the observed phenotypes. We also found that, as reported previously with docetaxel (34) and paclitaxel (36), genetic deficiency of OATP1B2 in mice is associated with decreased uptake of vincristine in organs that express OATP1B2, including the liver and whole DRG samples as measured by liquid chromatography with tandem mass spectrometry (LC/MS/MS) or matrix-assisted laser desorption/ionization–MS (MALDI-MS) imaging (Supplemental Figure 4, C–G). In support of a functional involvement of OATP1B2 in VIPN, it is noteworthy to point out that dipyridamole, a drug with moderate inhibitory potency against OATP1B-type transporters (43), was recently found to decrease intracellular concentrations of vincristine in hiPSC-derived neurons and partially protected against VIPN in mice without causing changes in the AUC of vincristine (13).

### Association of OATP1B2 with MTA-associated allodynia.

We next hypothesized that the OATP1B2-mediated transport mechanism observed for vincristine might also be operational for other MTAs that have neurotoxic properties. To test this hypothesis, we directly compared potential neuroprotective effects associated with OATP1B2 deficiency for clinically relevant doses of the microtubule-destabilizing agents vincristine, vinorelbine, and eribulin and the microtubule-stabilizing agents paclitaxel, docetaxel, and ixabepilone. Of the 6 MTAs tested, protection against treatment-related mechanical allodynia was only observed for paclitaxel and vincristine (Supplemental Figures 5 and 6). Although the behavioral methods used to study nociception here are somewhat subjective and warrant additional investigation to evaluate nonstimulus evoked nociception, such as place preference, the observations with MTAs suggest that the mechanism by which eribulin, vinorelbine, docetaxel, and ixabepilone gain access to peripheral nerves as an important site of injury may occur independently of OATP1B2 (Supplemental Figures 5 and 6 and Supplemental Table 2). Therefore, our subsequent studies focused exclusively on VIPN.

### Identification of OATP1B-type transport inhibitors.

**Figure 2:**
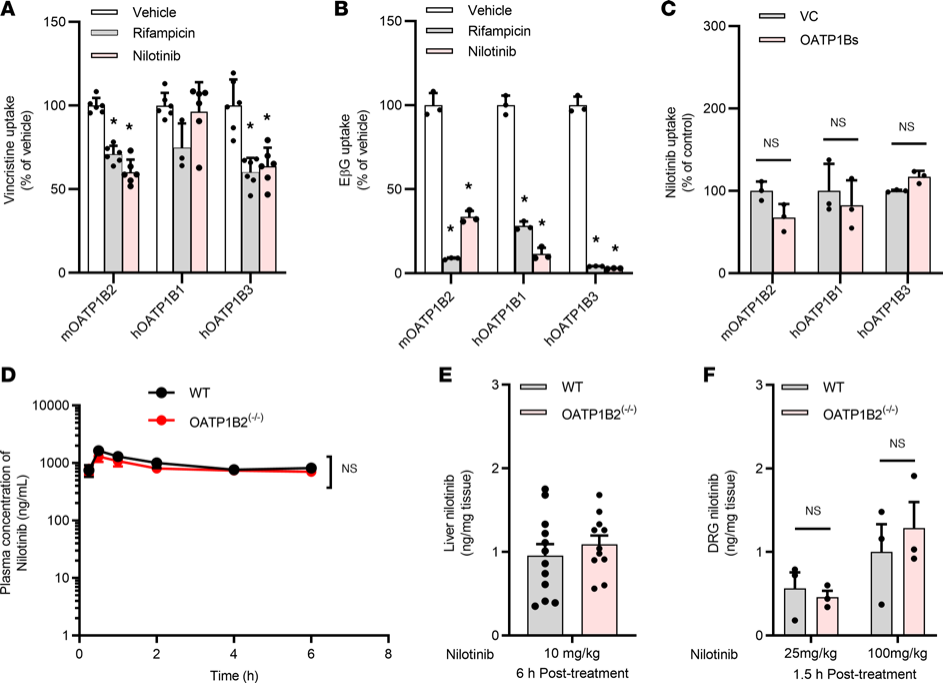
Inhibition of OATP1B-type transporters by nilotinib. (**A** and **B**) Nilotinib-mediated inhibition of vincristine and estradiol-17β-ᴅ-glucuronide (EβG) uptake in HEK293 cells overexpressing vector control, mouse (m) OATP1B2, human (h) OATP1B1, or hOATP1B3. Vincristine and EβG uptake data are expressed as percentage change compared with empty vector controls (*n* = 3–6 per group). Statistical analysis was performed using 1-way ANOVA with Dunnett’s post hoc test. * *P* < 0.05. (**C**) Nilotinib uptake in HEK293 cells overexpressing vector control, mOATP1B2, hOATP1B1, or hOATP1B3. (**D**) Plasma concentration-time profile of nilotinib (10 mg/kg) in male and female WT mice or OATP1B2^–/–^ mice (*n* = 12 per group, 6 for each sex). (**E**) Levels of nilotinib (10 mg/kg, 6 hours after treatment) in liver samples from male and female WT mice or OATP1B2^–/–^ mice (*n* = 12 per group, 6 for each sex). (**F**) Levels of nilotinib (25 mg/kg or 100 mg/kg, 1.5 hours after treatment) in DRG samples from female WT mice or OATP1B2^–/–^ mice (*n* = 3 per group). Statistical analysis was performed using an unpaired 2-tailed Student’s *t* test with Welch’s correction for liver and DRG drug accumulation. Data are shown as mean ± SEM.

Agents with inhibitory properties toward OATP1B-type transporters that could be exploited as neuroprotectants in conjunction with vincristine would ideally have (a) high potency, (b) high specificity, (c) low drug-to-drug interaction potential, (d) intrinsic antitumor properties, (e) favorable pharmaceutical properties, and (f) a mild and nonoverlapping toxicity profile. We hypothesized that the class of tyrosine kinase inhibitors (TKIs) is of particular interest in this context, as these agents have many of the above features, and several members of the class are known to potently inhibit the function of the related transporter OATP1B1 in vitro (54). In a recently reported screen of FDA-approved TKIs, we found that nilotinib, an inhibitor of the Bcr-Abl tyrosine kinase used for the treatment of patients with chronic myeloid leukemia, was the most potent inhibitor of OATP1B2 and OATP1B3 (55), in line with several recent reports (54, 56). Importantly, nilotinib was found to inhibit OATP1B-mediated transport at concentrations that can be achieved clinically (IC_50_, ~1 μM) (24). Since inhibition of OATP1B-mediated transport can be dependent on selection of the test substrate (57), we verified that nilotinib can also inhibit the OATP1B3-mediated uptake of both vincristine (Figure 2A) and other known substrates such as estradiol-17β-ᴅ-glucuronide (EβG) (Figure 2B and Supplemental Figure 7A) with a potency similar to that observed for the prototypical inhibitor rifampin. Nilotinib was found to be itself a poor substrate of OATP1B2 and OATP1B3 (Figure 2C), as suggested previously (56, 58), and consistently, its levels in plasma (Figure 2D and Supplemental Figure 7B) and liver (Figure 2E and Supplemental Figure 7C) were not influenced by OATP1B2 deficiency. We also verified that nilotinib can accumulate in DRG neurons of mice, independently of OATP1B2 genotype, in a dose-dependent manner (Figure 2F). This distribution property of nilotinib is likely a prerequisite for its OATP1B2-modulatory effects since the observed interactions are the result of a noncompetitive inhibitory mechanism. This supposition is consistent with our recent finding that the ability of nilotinib to inhibit OATP1B-type transport involves modulation of the Src-family kinase LYN, a known target of nilotinib that activates transport function by direct tyrosine phosphorylation (55). In line with the neuronal accumulation data, we found that the administration of nilotinib in mice is associated with significant inhibition of phosphorylated-LYN in DRG neurons (Supplemental Figure 8, A and B), suggesting that nilotinib has potential utility as a modulator of neuronal transport of vincristine and VIPN. The importance of LYN kinase in regulating OATP1B2 function and indirectly mediating vincristine transport is supported by the observation that genetic deficiency of LYN in mice provides complete protection against VIPN (Supplemental Figure 8C).

### OATP1B2 inhibition protects against VIPN.

**Figure 3:**
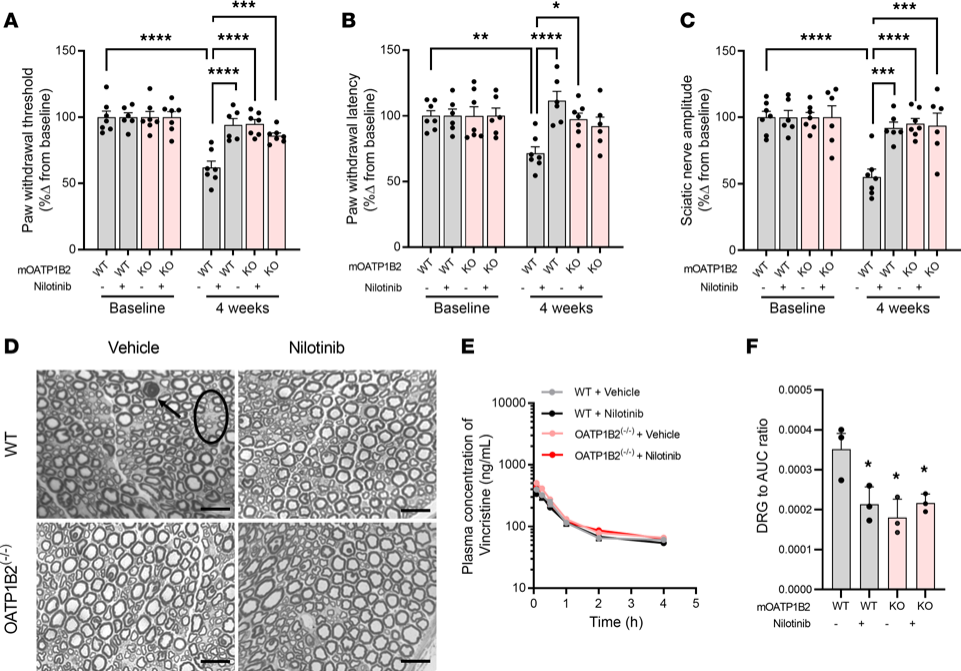
Inhibition of OATP1B2 with nilotinib protects against vincristine-induced peripheral neuropathy. (**A**–**C**) Mechanical allodynia (**A**), thermal hyperalgesia (**B**), and sciatic nerve maximal action potential amplitudes (**C**) at baseline and 4 weeks (*n* = 6–7 per group) in WT mice or OATP1B2-deficient (OAP1B2^–/–^) mice receiving weekly i.p. injections of vincristine at a dose of 1 mg/kg (cumulative dose 4 mg/kg). Mice were pretreated with vehicle (hydroxypropyl methylcellulose) or nilotinib (100 mg/kg; p.o.) 30 minutes before every vincristine injection. Statistical analysis was performed using a 2-way ANOVA with Tukey’s post hoc test. *P < 0.05, **P < 0.01, ***P < 0.001, ****P < 0.0001. (**D**) Light microscopy analysis of the sciatic nerve of WT mice and OATP1B2^–/–^ mice receiving weekly vincristine (cumulative dose 4 mg/kg). Mice were pretreated with vehicle (hydroxypropyl methylcellulose) or nilotinib (100 mg/kg; p.o.) 30 minutes before every vincristine injection. After 4 weeks, vincristine-induced axonopathy (arrow) and fiber loss (circle) was evident. Nilotinib pretreatment and OATP1B2 deficiency protected against these morphologic changes. Magnification, 63×. Scale bar:20 μm. (**E**) Plasma concentration-time profile of vincristine (1 mg/kg) in WT mice or OATP1B2^–/–^ mice pretreated with vehicle or nilotinib (100 mg/kg) (*n* = 7–9 per group). (**F**) DRG to plasma AUC ratio of vincristine (1 mg/kg) in WT mice or OATP1B2^–/–^ mice pretreated with vehicle or nilotinib (100 mg/kg) (*n* = 3 per group). Statistical analysis was performed using 1-way ANOVA with Dunnett’s post hoc test. *P < 0.05, compared with WT mice pretreated with vehicle. Data are shown as mean ± SEM.

Next, we evaluated whether pharmacological targeting of OATP1B2 preserves neuronal function after vincristine administration in a manner similar to that observed in OATP1B2^–/–^ mice. We found that chronic treatment of nilotinib (Supplemental Figure 9), administered orally at a dose of 100 mg/kg 30 minutes before each of 4 consecutive vincristine doses of 1 mg/kg, effectively protected against treatment-associated changes in mechanical allodynia (Figure 3A and Supplemental Figure 10, A and B), thermal hyperalgesia (Figure 3B), and sciatic nerve maximal AMP (Figure 3C and Supplemental Figure 11). Importantly, the protective effects associated with nilotinib pretreatment were dose dependent and could be replicated with pretreatment of orally administered rifampin, a known modulator of mouse OATP1B2 (59) and human OATP1B3 (60) that does not inhibit LYN kinase (Supplemental Figure 10C). These observations support the thesis that the neuroprotective properties of nilotinib in the context of VIPN are functionally dependent on inhibition of OATP1B2 function. Morphological evaluation showed that vincristine treatment was also associated with a reduction in cell size of DRG neurons in WT mice, and these morphologic abnormalities could be prevented by pretreatment with nilotinib or OATP1B2 deficiency (Supplemental Figure 12). Similarly, nilotinib pretreatment and OATP1B2 deficiency prevented vincristine-mediated axonal degeneration of sciatic and caudal nerves (Figure 3D and Supplemental Figure 13), as determined by the G-ratio, a measure of myelination and axonal integrity (Supplemental Figure 14). The lack of pronounced morphological changes observed for the group treated with single-agent vincristine may be due to the relatively low dose used in our studies. Importantly, while nilotinib pretreatment did not influence plasma levels of vincristine regardless of OATP1B2 genotype (Figure 3E and Supplemental Table 3), the accumulation of vincristine in whole DRG samples was significantly diminished by nilotinib (Figure 3F). These experiments shed light on the initiating mechanism responsible for VIPN and provide proof-of-principle that targeting of OATP1B-type transporters could represent an effective neuroprotective strategy. The notion that the CYP3A4/CYP3A5 inhibitor nilotinib (61) does not affect the plasma levels of vincristine and diminishes rather than increases VIPN indicates that pharmacological inhibition of OATP1B2 by nilotinib does not negatively influence the VIPN phenotypes through an influence on the metabolism of vincristine. These findings are consistent with the notion that the plasma pharmacokinetic profile of vincristine in mice is unaffected even by complete deficiency of all CYP3A isoforms (62). We are presently evaluating the existence of a direct relationship of VIPN with liver metabolism in CYP3A-KO mice with or without transgenic hepatic expression of CYP3A5, the main vincristine-metabolizing enzyme (63), to further substantiate the validity of our proposed intervention and the utility of mice as a predictive model organism.

### Nilotinib as an adjunct to vincristine therapy.

**Figure 4:**
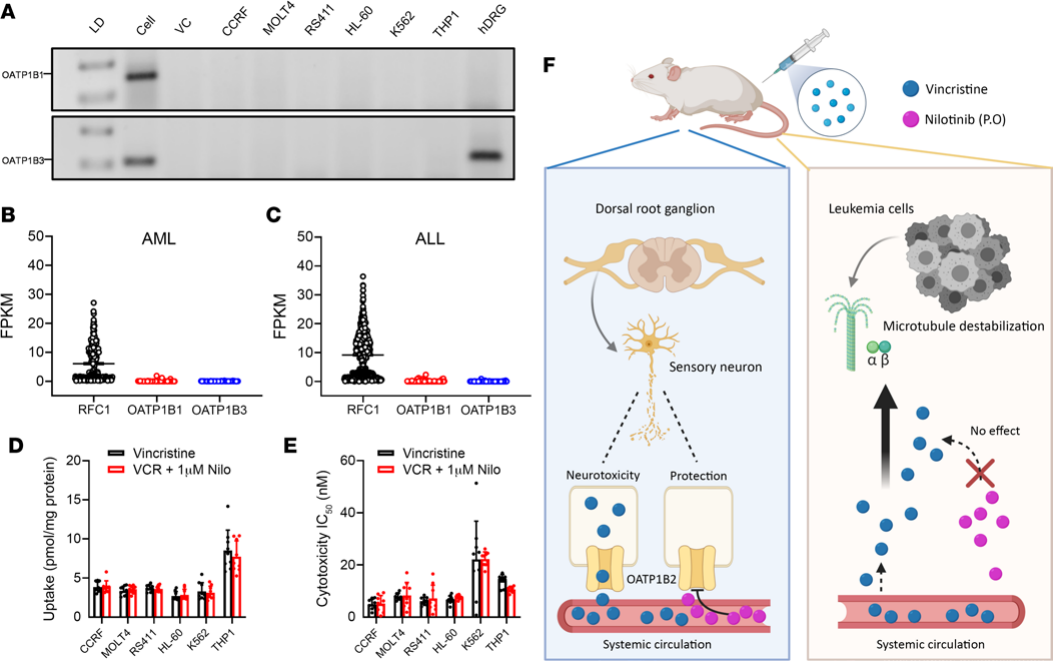
Nilotinib as an adjunct to vincristine therapy. (**A**) Expression of the human OATP1B1 and OATP1B3 genes in leukemia cells as measured by RT-PCR (depicted by the 154 bp product for OATP1B1 and 111 bp product for OATP1B3). HEK293 cells engineered to overexpress OATP1B1 or OATP1B3 were used as positive controls (denoted “Cell”), and cells transfected with an empty vector were used as negative controls (denoted “VC”). (**B** and **C**) Low OATP1B1 and OATP1B3 expression in malignant cells was confirmed in samples of 314 pediatric patients with acute myeloid leukemia (AML) (**B**) and 655 pediatric patients with B-lineage acute lymphoblastic leukemia (ALL) (**C**). Expression of the reduced folate carrier protein 1 (RFC1) was used as a reference gene in all samples. Median values in AML samples were RFC1 = 2.77, OATP1B1 = 0.00155, OATP1B3 = 0.00172; median values in ALL samples were RFC1 = 9.91, OATP1B1 = 0.00218, OATP1B3 = 0.00205. (**D**) Uptake of vincristine in leukemia cells in the presence or absence of nilotinib (1 μM) (*n* = 9 per group). Uptake data were normalized to total protein content. (**E**) Cytotoxicity of vincristine in leukemia cells in the presence or absence of nilotinib (1 μM). Cytotoxicity was measured by an MTT assay in 2-dimensional culture following continuous 72-hour exposure to vincristine (*n* = 9 per group). Data are shown as mean ± SEM. Statistical analysis was performed using a Student’s *t* test with Welch’s correction. (**F**) Proposed model of vincristine-induced injury to the peripheral nervous system in mice. Vincristine is taken up into cells within the peripheral nervous system by the transporter OATP1B2, ultimately leading to peripheral neuropathy, and these effects can be prevented by the OATP1B2 inhibitor nilotinib without negatively affecting anti-tumor efficacy.

Although combining vincristine with OATP1B-type transport inhibitors such as nilotinib could possibly reduce the incidence and severity of VIPN, it is important to establish that the antileukemic efficacy of the former is not compromised by the latter. The success of such a combination therapy would depend on the selected dosing/scheduling strategy and on the expression status of OATP1B3 in malignant cells. To gain preliminary insights, we previously evaluated the transcriptional profiles of OATP1B1 and OATP1B3 in human tumor specimens using normalized RNA-Seq data from 29 individual cancer cohorts in The Cancer Genome Atlas. This analysis indicated that these transporters are expressed at low levels in samples associated with the main vincristine indications (24). Consistent with this supposition, and with our previous findings in the NC-I60 panel of cell lines (64), we found that neither the OATP1B1 nor the OATP1B3 gene is detectable in 6 different leukemia cells lines (Figure 4A and Supplemental Table 4) or in samples from pediatric patients with leukemia (Figure 4, B and C), and that the uptake of vincristine in malignant cells is not substantially altered by nilotinib (Figure 4D). In addition, the translational potential of an OATP1B3 inhibitor–based intervention strategy to prevent VIPN is supported by our observation that nilotinib does not antagonize the cytotoxic effects of vincristine against preclinical models of leukemia (Figure 4, E and F). We further confirmed that 2 murine leukemia cell lines that are known to avidly accumulate vincristine by an unknown mechanism (27) express the OATP1B2 gene at very low levels and that nilotinib did not influence the uptake of vincristine into those cells (Supplemental Figure 15).

### Identification of DRG-specific biomarkers of OATP1B2 function.

To demonstrate direct modulation of neuronal OATP1B2 function following the administration of pharmacological inhibitors, identification of novel biomarkers is required to guide the selection of optimal doses and schedules to be used in conjunction with vincristine. Our preliminary data indicate that the plasma pharmacokinetic profile of vincristine is unaffected by genetic or pharmacological inhibition of OATP1B2, and this is consistent with the known limited contribution of this transporter to the elimination of other neurotoxic MTAs (36, 46). This finding also suggests that plasma levels of vincristine are not predictive of levels in the DRG neurons and cannot serve as proper biomarkers. As a suitably predictive alternative to plasma levels, we first considered the possibility that inherited genetic variability in the human OATP1B3 gene may predispose to an altered susceptibility to VIPN. This hypothesis would be consistent with our own functional studies (Supplemental Figure 1A) and with prior reports indicating that variants in drug transporter genes have been associated with interindividual differences in the incidence and severity of adverse events following treatment with substrate drugs (65). In a cohort of pediatric patients with acute lymphoblastic leukemia receiving treatment with vincristine (66), however, we found that common OATP1B3 variants were not statistically significantly associated with VIPN (Supplemental Table 5), suggesting that genetic approaches may be less suitable for the optimization of preventative treatment strategies in this context.

**Figure 5:**
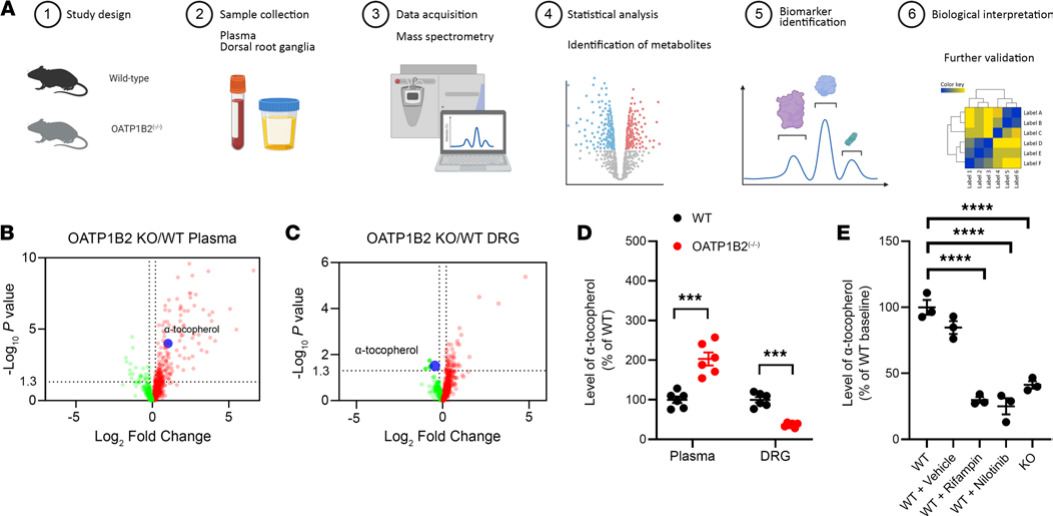
Identification of a DRG-specific endogenous biomarker of OATP1B2. (**A**) Schematic overview of the untargeted metabolomics screen to discover an endogenous biomarker of OATP1B2. (**B**) Volcano plot of differentially endogenous metabolites in untreated plasma of WT mice or OATP1B2-deficient (OATP1B2^–/–^) mice. Positive fold change indicates higher plasma concentration in OATP1B2^–/–^ mice (*n* = 6 per group). Dotted lines indicate a log_10_ *P* value threshold of > 1.3 and a log_2_FC (fold change) of ± 0.2. (**C**) Volcano plot of differentially endogenous metabolites in untreated whole DRG samples of WT mice or OATP1B2^–/–^ mice. Negative fold change indicates lower DRG levels in OATP1B2^–/–^ mice (*n* = 6 per group). (**D**) Plasma levels and DRG/plasma ratios of α-tocopherol in WT mice or OATP1B2^–/–^ mice in secondary validation studies. Statistical analysis was performed using an unpaired 2-tailed Student’s *t* test with Welch’s correction. ***P < 0.001 (*n* = 6 per group). (**E**) Relative DRG/plasma ratio of α-tocopherol in WT mice or OATP1B2^–/–^ mice 2 hours after the administration of vehicle control (hydroxypropyl methylcellulose), rifampin (20 mg/kg; i.p.), or nilotinib (100 mg/kg; p.o.). Data are normalized to baseline levels observed in WT mice (*n* = 3 per group). Statistical analysis was performed using 1-way ANOVA with Dunnett’s post hoc test. ****P < 0.0001.

Next, we hypothesized that biomarkers of neuronal OATP1B-type transport function can be identified by probing naturally occurring metabolites (“endogenites”) that are transported by neuronal OATP1B2 and that can be detected in plasma. To identify endogenites of interest, we applied a nontargeted metabolomics platform to plasma and DRG specimens from WT mice and OATP1B2^–/–^ mice (Figure 5A) (67) and identified α-tocopherol, the most prevalent form of vitamin E, as a DRG-specific biomarker of OATP1B2 (Figure 5, B and C). This finding was independently verified (Figure 5D and Supplemental Tables 6 and 7), and we confirmed that α-tocopherol is a transported substrate of OATP1B3 that can mediate inhibition of transporter-mediated uptake of EβG and vincristine in vitro (Supplemental Figure 16, A–C). Furthermore, we found that the DRG/plasma concentration ratio of α-tocopherol was sensitive to inhibition by pretreatment with rifampin and nilotinib at clinically relevant doses to a degree that is similar to that observed at baseline in OATP1B2^–/–^ mice (Figure 5E and Supplemental Figure 16D). These findings suggest that plasma levels of α-tocopherol serve as a bona fide biomarker of neuronal OATP1B-type transport function that may have utility in future validation studies aimed at evaluating the dose dependence and time dependence of transport modulation by novel inhibitors, and at refining the dose and schedule of such inhibitors in patients undergoing treatment with vincristine to ameliorate VIPN.

## Discussion

In the current study, we identified OATP1B-type transporters as mediators of vincristine uptake and demonstrated that this transport mechanism is a prerequisite for vincristine-induced injury to the peripheral nervous system. In particular, we found that genetic deficiency of the murine transporter OATP1B2 is associated with complete protection against VIPN and that this phenotype could not be reversed by transgenic hepatic expression of the orthologous human transporter, OATP1B3. Furthermore, we found that OATP1B2 can be targeted pharmacologically with multiple FDA-approved agents, including the TKI nilotinib, and that pretreatment with such agents can offer neuroprotection against VIPN without influencing the plasma levels and antitumor properties of vincristine. In addition, α-tocopherol was identified as a biomarker of neuronal OATP1B-type transporter function, and this endogenous compound can potentially serve as a companion diagnostic to optimize combinatorial regimens of OATP1B modulators and vincristine to prevent VIPN.

Vincristine belongs to the class of MTAs that can be classified into 2 main groups, the microtubule-destabilizing agents and the microtubule-stabilizing agents. The destabilizing agents inhibit microtubule polymerization and include the *Vinca* alkaloids (e.g., vincristine and vinorelbine) and the halichondrins (e.g., eribulin), while the stabilizing agents enhance microtubule polymerization and include the taxanes (e.g., paclitaxel and docetaxel) and the epothilones (e.g., ixabepilone). Although the clinical use of most MTAs is associated with peripheral neurotoxicity, the incidence and severity of this side effect is particularly problematic with vincristine. While various preventative approaches have been evaluated to prevent and/or treat VIPN (68, 69) — for example, by pretreatment with the tricyclic antidepressant amitriptyline or the synthetic neurotrophic peptide Org 2766 — the results of these efforts have been largely contradictory to the hypothesis (70). Furthermore, the translational exploration of many of the previously proposed intervention strategies has been hampered by the recognition that (a) vincristine has multiple intracellular targets, and hence, blocking a single injurious event will only have partial protective effects; and (b) the protective approach may diminish the antileukemic effects of vincristine, given the potential overlap in cell death signaling pathways between normal cells and malignant cells (8). Therefore, an ideal approach is to simultaneously protect the peripheral nerves against vincristine without affecting the therapeutic effects against cancer. The development of such an approach would rely on the identification of the critical differences between normal and malignant cells that drive toxic responses to vincristine.

Recent studies have detailed the neuroinflammatory mechanisms leading to VIPN in mice and suggested that repurposing of the IL-1 receptor antagonist anakinra (Kineret) may be an effective cotreatment strategy to prevent VIPN without adversely affecting the efficacy of vincristine in murine models of medulloblastoma (52). This work originated from the notion that vincristine induces a striking upregulation of inflammatory genes in DRG neurons and release of proinflammatory cytokines and chemokines, including IL-1β, TNF-α, IL-6, and CCL2 (71). These observations are consistent with previous work suggesting that the severity of neurotoxic effects associated with MTAs such as vincristine is directly related to the levels at which they accumulate in DRG neurons within the peripheral nervous system (17) and with our present finding that this distribution property is dependent on OATP1B-mediated transport.

In past studies evaluating the transmembrane transport of vincristine, it was asserted that the predominant mechanism of accumulation is associated with phospholipid bilayer transport (passive diffusion) (72). More recent research (73), however, has led to the recognition that the cellular uptake of even fairly large and hydrophobic plant alkaloids such as *Vinca* alkaloids and taxanes is instead mediated by transporters (74). This thesis is in agreement with studies indicating that vincristine interacts with the human OATP1B1 and OATP1B3 transporters (23) and provides compelling support for ongoing efforts aimed at blocking these transporters, reducing intraneuronal concentrations of neurotoxic agents, and ultimately protecting against a dose-limiting injury (75). We previously reported that OATP1B2 is detectable in mouse DRG specimens (24), and our current investigation suggests that OATP1B3 fulfills the same function as a neuronal vincristine transporter in humans, as indicated by its high expression relative to OATP1B1 in pooled human DRG samples and the increased transport efficiency of vincristine in OATP1B3-transfected cells compared with cells engineered to overexpress OATP1B1 (23). It should be pointed out that, since the expression of OATP1B-type transporters was determined using whole DRG samples, a contribution of these transporters expressed in nonneuronal cell types, such as macrophages, cannot not be entirely excluded without a neuron-specific OATP1B2-KO model. Ongoing studies are focused on the development of such models and on documenting neuropathy and its rescue by intraepidermal nerve fiber density measurement and axon counts in the nerve.

We found that OATP1B2 deficiency in mice is associated with complete protection against VIPN, an observation that provides direct empirical evidence for the notion that vincristine is not able to diffuse through an unhindered phospholipid bilayer existing in intact biological membranes. This conclusion is further consistent with the finding that the neuroprotection observed in OATP1B2-deficient mice could be phenocopied in WT mice pretreated with various structurally and functionally diverse OATP1B2 inhibitors, including the TKI nilotinib. We recently reported that TKIs affect OATP1B-type transporters via a noncompetitive mechanism that involves inhibition of LYN-kinase–mediated phosphorylation (55), and this is consistent with our present findings that the administration of nilotinib in mice impairs tyrosine phosphorylation of LYN in DRG neurons and that LYN deficiency protects against VIPN. Based on the high degree of sequence homology between mouse OATP1B2 and human OATP1B3, their similar tissue localization, and largely overlapping substrate and inhibitor specificity (76), we expect that nilotinib is also able to inhibit the function of LYN in human DRG neurons. This thesis is consistent with a recent report suggesting that potent inhibition by nilotinib of OATP1B-type transporters in in vitro models occurs at concentrations that have potential in vivo significance (55).

Compared with other TKIs with modulatory properties toward OATP1B-type transporters, nilotinib has pharmaceutical and pharmacological features that suggest it might be an excellent modulator of VIPN. These include a relatively high oral bioavailability, slow systemic clearance, and a long half-life (77), thus ensuring that sufficiently high and persistent local drug levels can be achieved even after single oral doses. Although TKIs such as nilotinib are typically administered on a daily basis for prolonged durations, it is noteworthy that the use of high-dose, pulse-exposure dosing is becoming an increasingly frequently applied concept in oncology (78), and the clinical experience with such intermittent strategies will ultimately allow easy translation of our proposed concept to use nilotinib as a transporter inhibitor in conjunction with vincristine-based chemotherapy in patients. We acknowledge that, while most side effects associated with nilotinib are mild, reversible, and easily managed, the drug’s prescribing information carries a black box warning for QT prolongation (79). It should be pointed out, however, that the median time from the start of nilotinib therapy using a conventional chronic regimen (i.e., once or twice daily dosing without interruption) to the onset of such QT events is > 14 months (range, 2–68 months) (80). In our studies, we aimed to interrogate the response to the nilotinib-vincristine combination following acute or intermittent exposure to the TKI. Therefore, we anticipated that nilotinib will not be intrinsically cardiotoxic in such combination regimens.

Importantly, we found that nilotinib did not antagonize the antileukemic properties of vincristine in various preclinical models, and this suggests that vincristine can be taken up into leukemic cells by an unknown mechanism that is insensitive to nilotinib-mediated inhibition and that is operational independently of OATP1B-type transporters. This observation supports the possibility that, in humans, OATP1B3 represents a selective transporter that, when inhibited, reduces cellular injury to neurons without altering the treatment efficacy of vincristine against leukemias. The observations made here with nilotinib-vincristine combinations in leukemia cells are in line with previously reported synergistic in vitro effects of nilotinib and vincristine in certain ABCB1- or ABCC10-overexpressing tumors (81–83), and with the absence of antagonism in several other preclinical tumor models, including oral squamous cell carcinoma (84, 85). Importantly, genetic deficiency or inhibition of uptake transporters mediating tissue uptake of cancer drugs does not necessarily cause simultaneous changes in measures of systemic exposure to substrates drugs (24, 36, 46, 86). In this context, it is worth mentioning that the feasibility of adding nilotinib to high-dose, vincristine-based induction chemotherapy in adult patients with newly diagnosed Philadelphia chromosome–positive acute lymphoblastic leukemia has been reported and was shown to achieve high complete molecular remission and 2-year hematologic relapse-free survival rates (87). Although additional studies are required to confirm our findings in properly designed model systems and additional vincristine-sensitive leukemias, including cell line– and patient-derived xenograft models in which transporter inhibitors are given intermittently before each vincristine dose, these observations indicate that combining vincristine with agents such as nilotinib has the potential to simultaneously reduce toxicities without negatively influencing the overall antileukemic effects.

The translational potential of our proposed intervention concept to prevent VIPN is supported by the identification of α-tocopherol as a bona fide DRG-specific endogenous biomarker of OATP1B-type transporter function that can be measured in the circulation. While α-tocopherol is well known to affect many biological functions, such as immune response and blood clotting, its connection to neuronal transport activity has not been previously documented and provides a rationale for its future usage as a biomarker. This finding is consistent with a wealth of literature indicating that many endogenous substances exhibit transporter-dependent tissue distribution properties and that their concentrations in plasma are altered when the activity of pertinent transporters is modulated (88–90). Indeed, transporter biomarkers are now routinely monitored in early-phase dose-escalation trials where changes in their exposure upon comedication with a drug candidate can predict the risk for potential transporter-mediated drug-to-drug interaction liabilities (91). Several endogenous compounds, including coproporphyrins and sulfated or glucuronidated bile acids, were previously identified as biomarkers OATP1B-mediated hepatic transport and have provided mechanistic insight into pharmacokinetic drug-to-drug interactions (92–94). In our global untargeted metabolomics screen of DRG samples from WT mice and OATP1B2-deficient mice, none of the coproporphyrins and conjugated bile acids were identified as DRG-specific biomarkers of OATP1B2 function. The thesis that α-tocopherol can serve this role in both mice and humans is consistent with prior studies indicating that (a) xenobiotics with structural similarity to α-tocopherol, such as the thiazolidinedione insulin sensitizer drug troglitazone (95), are transported substrates of OATP1B3 (96); (b) up to 90% of total body mass of α-tocopherol is recovered in the liver where OATP1B3 is highly expressed (97); and (c) α-tocopherol is detectable at high levels in the peripheral nervous system of human subjects but not in those with vitamin E deficiency (98). It is further worth noting that α-tocopherol supplementation has been explored as a strategy to prevent peripheral neurotoxicity induced by various chemotherapeutic drugs, including taxanes (99, 100). In addition, vitamin E–based formulations have been developed for various anticancer drugs with the goal to improve aqueous solubility of hydrophobic drugs and enhance therapeutic efficiency as well as offer neuroprotection (101–103). Further study is required to determine to what extent the ability of α-tocopherol to inhibit OATP1B2- and OATP1B3-mediated transport contributes to the claimed neuroprotective activity associated with vitamin E supplementation in patients with cancer.

In conclusion, we identified a previously unrecognized pathway of VIPN that is mediated by OATP1B-type transporters. The function of this transport system is sensitive to pharmacological inhibition by various prescription drugs and can prevent VIPN without compromising the anticancer properties of vincristine in multiple models of leukemia. In addition, we identified α-tocopherol as a DRG-specific OATP1B-type transporter biomarker that could serve as a companion diagnostic to guide dose selection of pharmacological inhibitors in the future development of combinatorial regimens with vincristine. These findings shed light on the fundamental basis of VIPN and provide a rationale for the future development of an intervention strategy using transporter inhibitors to mitigate a debilitating side effect associated with vincristine.

## Methods

Supplemental Methods are available online with this article.

### Animal studies.

All animals were housed in a temperature-controlled environment with a 12-hour light cycle and were given a standard chow diet and water ad libitum. For all experiments, age- and sex-matched WT mice or transporter-deficient mice (8–12 weeks) were used. Detailed information regarding sources and origins of the rodent models is provided in the Supplemental Methods.

### Statistics.

Data presented represent the mean ± SEM before and/or after normalization to baseline values and are expressed as a percentage unless stated otherwise. All experiments were performed using multiple replicates and were performed independently on at least 2 separate occasions. An unpaired 2-tailed Student’s *t* test with Welch’s correction was used for comparisons between 2 groups, and a 1-way ANOVA with Dunnett’s post hoc test was used for comparing more than 2 groups. Behavioral data were analyzed using 2-way ANOVA with Tukey’s or Bonferroni’s post hoc test across time points and groups. *P* < 0.05 was considered significant.

### Study approval.

All animals were handled according to and approved by the University Laboratory Animal Resources (ULAR) Animal Care and Use Committee at The Ohio State University, under an approved protocol (no. 2015A00000101-R2).

### Data availability.

The data that support the findings of this study are available on request from the corresponding author; see Supplemental Data Values.

## Author contributions

AS and SH conceived the study; YL, AS, and SH designed research; YL, TD, MN, AFL, AAG, YJ, WY, KMH, MEU, MC, DFD, XC, SR, and YW performed research; RHH, AAG, YJ, JRT, DMM, ASM, MBL, YW, ABH, WEE, SDB, and GC contributed new reagents and analytic tools; YL, TD, WY, AS, and SH analyzed data; and YL, AS, and SH wrote the paper.

## Supplementary Material

Supplemental data

Supporting data values

## Version 1 06/22/2023

In-Press Preview

## Version 2 07/24/2023

Electronic publication
