# Supplementary material for: Targeting a xenobiotic transporter to ameliorate vincristine-induced sensory neuropathy
Source: JCI Insight. 2023 Jul 24;8(14):e164646. doi: 10.1172/jci.insight.164646 (PMC10443802; doi:10.1172/jci.insight.164646)
Supplement: Supplemental data [file jciinsight-8-164646-s165.pdf]

Supplementary Information for

Targeting a xenobiotic transporter to ameliorate vincristine-induced sensory neuropathy

Yang Li[1,10], Thomas Drabison[1], Mahesh Nepal[1,10], Richard H. Ho[2], Alix F. Leblanc[1], Alice A. Gibson[1], Yan Jin[1], Wenjian Yang[3], Kevin M. Huang[1], Muhammad Erfan Uddin[1], Mingqing Chen[1], Duncan F. DiGiacomo[1], Xihui Chen[1], Sobia Razzaq[1], Jeffrey R. Tonniges[4], Dana M. McTigue[5], Alice S. Mims[6], Maryam B. Lustberg[7], Yijia Wang[8], Amanda B. Hummon[8], William E. Evans[3], Sharyn D. Baker[1], Guido Cavaletti[9], Alex Sparreboom[1], and Shuiying Hu[1,10]

[1]Division of Pharmaceutics and Pharmacology, College of Pharmacy & Comprehensive Cancer Center, The Ohio State University, Columbus, OH 43210; [2]Vanderbilt University Medical Center, Nashville, TN 37240; [3]Department of Pharmaceutical Sciences, St. Jude Children's Research Hospital, Memphis, TN 38105; [4]Campus Microscopy and Imaging Facility, The Ohio State University, Columbus, OH 43210; [5]The Belford Center for Spinal Cord Injury & Department of Neuroscience, College of Medicine, The Ohio State University, Columbus, OH 43210; [6]Division of Hematology, Department of Internal Medicine, The Ohio State University, Columbus, OH 43210; [7]The Breast Center at Smilow Cancer Hospital at Yale, New Haven, CT 06511; [8]Department of Chemistry and Biochemistry and the Comprehensive Cancer Center, The Ohio State University, Columbus, OH 43210; [9]Experimental Neurology Unit and Milan Center for Neuroscience, School of Medicine and Surgery, University of Milano-Bicocca, 20900 Monza, Italy; [10]Division of Outcomes and Translational Sciences, College of Pharmacy & Comprehensive Cancer Center, The Ohio State Un0versity, Columbus, OH 43210.

Corresponding author

Shuiying Hu, Division of Outcomes and Translational Sciences, College of Pharmacy & Comprehensive Cancer Center, The Ohio State University, 500 West 12th Avenue, Columbus, OH 43210. Tel: +1-614-685-8028; Fax: +1-614-688-4028; Email: hu.1333@osu.edu

Supplemental Information Text

Detailed Materials and Methods

Animals. For all experiments, age- and sex-matched mice (8-12 weeks old) were used. All animals were housed in a temperature-controlled environment with a 12-h light cycle, given a standard chow diet and water *ad libitum*, and were handled according to the University Laboratory Animal Resources Animal Care and Use Committee at The Ohio State University, under an approved protocol (2015A00000101-R2). Wild-type mice on a DBA/1LacJ or FVB background were purchased from The Jackson Laboratory or Taconic Biosciences. OATP1B2-deficient [OATP1B2(-/-)] mice on a DBA/1LacJ background were provided by Drs. Richard B. Kim (Western University, London, Ontario, Canada) and Jeffrey L. Stock (Pfizer, Groton, CT). OATP1A/1B-deficient [OATP1A/1B(-/-)] mice were purchased from Taconic Biosciences (Taconic Biosciences, Cambridge City, IN). LYN-deficient [LYN(-/-)] mice were kindly provided by Dr. Clifford A. Lowell (University of California, San Francisco, CA) (1).

Peripheral neuropathy assessment. Mice received a single i.p. injection of vincristine diluted in normal saline at a dose of 1 mg/kg. The nocifensive behaviors (hind paw flinching) after mechanical stimulation (Von Frey Hairs test, VFH) were used to assess the development of mechanical allodynia before and at 24, 48, 72 h and 1 week after drug administration. Mice were individually housed and acclimated to handling and to the VFH testing apparatus for several days prior to start of the experiment. Baseline measurements were obtained prior to dosing. Behavioral testing was conducted in a quiet room following procedures previously described (2-4). Briefly, mice were placed on a wire mesh floor while loosely restrained in a plexiglass compartment and allowed to acclimate for 1 h prior to initiation of testing. For testing, a blunt filament (0.5 mm diameter) was applied perpendicularly to the plantar surface of the hind paw with a constant force for 2-5 s. When a spontaneous hind paw withdrawal occurred, the stimulus was stopped and the gram force of the pressure to withdrawal was recorded. This procedure was assessed 3 times by applying the filament to the mid-plantar region of each hind paw from beneath the mesh floor and with intervening intervals of a few seconds. Values for each animal were then used to average across

groups to yield a mean group threshold response, an upper cutoff limit of 6 g was set to prevent tissue damage.

For studies evaluating chronic neurotoxicity, vincristine (1 mg/kg) was administered once a week by i.p. injection for four weeks (cumulative dose 4 mg/kg), preceded by nilotinib (100 mg/kg; p.o.) or its vehicle (0.5% methylcellulose in water). A clinical electro-diagnostic system (Ultra Pro S100, Natus Neurology) was used to perform nerve conduction studies (3). The supramaximal action potential amplitude (AMP) and the nerve conduction velocity (NCV) were measured for the caudal and sciatic nerve, 24 h before treatment (baseline) and at the end of the experiment. Stimulating electrodes were placed on the fourth digits of the hind paw, and two recording electrodes were placed 10 mm proximally, near the ankle. Similarly, stimulating electrodes were placed at the base of the tail, and two recording electrodes were placed 35 mm proximally on the tail. For both the digital and caudal nerve, a total of 10 supramaximal stimulations was delivered. Velocity was calculated as a ratio of the distance from stimulating electrodes divided by the distant latency, and amplitude from peak-to-peak. Mice were maintained under isoflurane anesthesia delivered via a nose cone and the body temperature was controlled during the recordings with a heating pad. AMP and NCV were normalized and expressed as a percent change from baseline to account for inter-experimental variability in baseline recordings. Thermal hyperalgesia (Hargreaves test) was evaluated with a dynamic esthesiometer (model 390, IITC Life Science) (3). Baseline measurements were obtained prior to dosing. On the day of testing, mice were placed in a Plexiglas chamber for 45 min to acclimate. The stimulus was stopped and the time to withdrawal latency was recorded. This was assessed alternately on each hind paw every 2 min for 3 consecutive trials to obtain a mean value of the heat tolerated by the mice. To prevent tissue damage, an upper cutoff limit of 30 s was set, after which the increasing thermal stimulus was terminated.

Gross motor performance associated with the tested vincristine regimens was evaluated by ladder tests and open field tests, as previously described (5, 6). Briefly, mice received a single i.p. injection of vincristine at a dose of 1 mg/kg. The ladder test and open field test were used to assess the development of motor neuron toxicity before and at 24, 48, and 72 h after drug administration. For elevated ladder tests, the elevated ladder was placed 30 cm above the ground and made up of two clear side walls (1 m long and 19 cm high) and a cover on the top and several metal rungs (3 mm diameter),

where the distance between two adjacent rungs is changeable (1-5 cm). The elevated ladder tests were initiated after mice had been acclimated to their surrounding for at least 4 h. The mice were placed at one end of the ladder and encouraged to move forward by a food reward at the other end of the ladder. If a mouse had a misstep, their foot contacts the metal plate, completes a circuit, and is automatically counted by an electrical counter. Experiments were repeated 3 times for each mouse, and an average of the three trials was used for analysis. Open-field locomotion was assessed with the Basso Mouse Scale (BMS) rating system (7). The open field was a round table that was 20.32 cm high and 1.524 m in diameter situated in a quiet testing room with normal lighting. The mice were acclimated to the testing table for 20 min daily for a week before drug administration. Two analysts blinded to the composition of the treatment groups simultaneously scored hindlimb locomotion for 4 min per mouse using a score sheet described previously (7). Parameters of interest included paw position (rotated in, rotated out, parallel placement), trunk stability, tail position, coordination, and plantar and dorsal support. Quantification was based on a scoring system where conditional variables are given a numerical representation in each category. A higher number correlates to better hindlimb locomotor function and 'normal' behavior scores a 9; conversely, a lower number represents abnormal locomotion.

Pharmacokinetic studies. Pharmacokinetic studies were performed following an established protocol (8). To determine the plasma concentration-time profiles of nilotinib in mice, oral nilotinib (formulated in 0.5% hydroxypropyl-methylcellulose in water) was administered at a dose of 10 mg/kg. Serial whole blood samples (0.25, 0.5, 1, 2, 4, and 6 h) were collected from the submandibular vein for the initial three time-points, from the retro-orbital sinus vein for the subsequent two time-points, and cardiac puncture at the terminal time-point. Blood samples were centrifuged at 11,000 rpm for 5 min, and the plasma supernatants collected and stored at -80°C until analysis by a validated method based on reversed-phase liquid chromatography coupled to tandem mass-spectrometric detection (LC-MS/MS). Livers were collected from the same animals at endpoint and, to prevent continuing metabolic activity, tissues were snap frozen using liquid nitrogen. In another experiment, nilotinib was administrated orally at doses of 25 mg/kg or 100 mg/kg, and after 1.5 h, plasma, liver, and whole DRG were collected. All plasma and tissue specimens were stored at -80°C until further processing.

The pharmacokinetic profiles of vincristine in mice were evaluated after administration by i.p. injection at a dose of 1 mg/kg. Whole blood samples were collected 0.08, 0.25, 0.5, 1, and 4 h, and livers were collected at the final time point. To evaluate the influence of nilotinib pre-treatment on the profile of vincristine, whole blood and liver samples were collected at the same time points. All plasma and tissue specimens were stored at -80°C until further processing. All tissues were homogenized with 5-mm stainless steel bead (Qiagen) and processed for 4 min at 40 Hz. Finally, the beads were removed, and the homogenized samples were stored at -80°C until analysis by LC-MS/MS.

Pharmacokinetic parameters were calculated by non-compartmental analysis using Phoenix WinNonlin version 8.0 (Certara). Peak plasma concentration (C_{max}) was determined by visual inspection of the data from the concentration-time curves. The linear trapezoidal rule was used to obtain the area under the plasma concentration-time curve (AUC), and drug levels in tissues were corrected for contaminating plasma.

Determination of drug levels. Nilotinib was quantified using a Vanquish UHPLC coupled with a Quantiva triple quadrupole mass spectrometer from Thermo Fisher Scientific was used for LC-MS/MS analysis. An Accucore aQ column (150 × 2.1 mm, dp = 2.6 µm, Thermo Fisher Scientific) was protected by a C18 AQUASIL guard cartridge (2.1 mm × 10 mm, dp =3 µm, Thermo Fisher Scientific). The injection volume was 5.0 µL. The temperature of the autosampler rack was 4°C, and the temperature of the column was maintained at 50°C. Mobile phase A consisted of water with 0.1% (v/v) formic acid and mobile phase B consisted of acetonitrile: methanol (1:1) with 0.1% (v/v) formic acid. The total run time was 4.6 min. The gradient conditions were as follows: 0–0.5 min, 45% B; 0.5–3.5 min, 45 to 90%, 3.5–4.0 min, 90% B;4.0–4.1 min, 90% to 45% B; 4.1–4.6 min, 45% B with a flow rate was 0.4 mL/min. The MS assay setting with the positive voltage applied to the ESI capillary was set at 4000 V, and the capillary temperatures was 375 °C with a vaporizer temperature of 450 °C. Argon was used as the collision gas at a pressure of 1.5 mTorr. Precursor molecular ions and product ions were recorded for confirmation and detection of nilotinib (530.162→289.025), using [$^{2}H_{3}$]-nilotinib (nilotinib-d3) as an internal standard (533.1→288.94). Results from assay validation studies revealed that the within-day precision and

between-day precision ranged 0.61-3.43%, and the accuracy ranged 96.9-103%. The lower limit of quantification was 5 ng/mL.

Vincristine was quantified using the same equipment and an Accucore aQ column (50 × 2.1 mm, dp = 2.6 µm, Thermo Fisher Scientific) protected by a C18 AQUASIL guard cartridge (2.1 mm × 10 mm, dp =3 µm, Thermo Fisher Scientific). The injection volume was 2.0 µL. The temperature of the autosampler rack was 4°C, and the temperature of the column was maintained at 50°C. Mobile phase A consisted of water with 0.1% (v/v) formic acid and mobile phase B consisted of acetonitrile with 0.1% (v/v) formic acid. The total run time was 2.2 min. The gradient conditions were as follows: 0–0.5 min, 10% B; 0.5–0.51 min, 10 to 50%, 0.51–1.8 min, 50% to 95% B; 1.8–1.81 min, 95% to 10% B; 1.81–2.2 min, 10% B with a flow rate was 0.4 mL/min. The MS assay setting with the positive voltage applied to the ESI capillary was set at 3536.36 V, and the capillary temperatures was 375°C with a vaporizer temperature of 450 °C. Argon was used as the collision gas at a pressure of 1.5 mTorr. Precursor molecular ions and product ions were recorded for confirmation and detection of vincristine (825.438→765.354), using [$^{2}H_3$]-vincristine sulfate (vincristine-d3) as an internal standard (828.488→768.275). Results from assay validation studies revealed that the within-day precision and between-day precision ranged 1.87-5.20%, and the accuracy ranged 91.7-107%. The lower limit of quantification was 2.5 ng/mL.

Vincristine levels in whole DRG samples (L4 and L5) from wild-type and OATP1B2(-/-) mice were measured by MALDI Mass spectrometry imaging at 30 min, after i.v. administration of vincristine at a dose of 4 mg/kg. DRG tissues were embedded in gelatin and cryosectioned to 12 µm thick. The sample slides were coated with 10 mg/mL of 2,5-dihydroxybenzoic acid (DHB), with 16 passes at a 100 µL/min flow rate and were imaged with a laser spot size of 50 µm. Imaging data were acquired with a Bruker 15T FTICR using SciLS Lab Version 2022a Pro. All the data were normalized by total ion count of the imaged samples (9).

Morphologic and morphometric evaluation. For morphologic evaluation, L4-L5 DRGs were fixed by immersion in 4% paraformaldehyde and 2% glutaraldehyde, and sciatic and caudal nerve were fixed by immersion in 2.5% glutaraldehyde. Samples were post fixed in OsO_4, embedded in epoxy resin, and used for light microscopy and morphometric analysis. Semi-thin 1-µm sections (spaced at 50-µm intervals) of

DRG, sciatic and caudal nerves were prepared, stained with toluidine blue, and examined with a Zeiss Axioskop WideField Light Microscope, as described previously (10). Representative images were captured with a QIClick Digital CCD color camera. For DRG morphometrics, the somatic size was measured for each cell containing nucleolus, and at least 200 DRG neurons per animal were manually measured and analyzed with a computer-assisted image analyzer (ImageJ software, Bethesda, MD). For sciatic nerve, all myelinated fibers evaluable in the analyzed space were counted and the internal (axonal) and external (total) diameters of myelinated fibers were measured on at least 500 myelinated fibers/nerve. The histograms of g-ratio (axonal diameter/whole fiber diameter as a measure of myelination degree in each fiber) distribution were generated. An observer blinded to the group compositions performed all the morphometric determinations.

Cellular accumulation. Human embryonic kidney (HEK293) cells (ATCC) overexpressing OATP1B1, OATP1B3, or OATP1B2 were generated as described previously (11). The transporter nomenclature used throughout is based on recent recommendations proposed by Hagenbuch and Stieger (12). The transporter-expressing cells were functionally characterized by assessing the uptake of the prototypical substrate, estradiol-17β-ᴅ-glucuronide (EβG) (0.2 μM). HEK293 cells were maintained in DMEM supplemented with 10% heat-inactivated FBS, and routinely checked to ensure there was no mycoplasma contamination (MycoAlert Detection Kit). Passages were kept to a minimum and cells were not used beyond passage 30. The cell culture conditions and details of accumulation experiments for tritium-labeled vincristine (ART 03310) were performed as described previously (11). Briefly, prior to uptake experiments, cells were grown to 90% confluence on poly-lysine coated multi-well plates. For uptake, cells were briefly rinsed with warm 1X PBS and incubated in the presence or absence of a vehicle or inhibitor, prepared in serum and phenol-red free DMEM for 15 min (pre-treatment). The pre-treatment was rinsed off with warm 1X PBS followed by the addition of radiolabeled compounds for 5-30 minutes. After incubation, transport was halted by aspirating radiolabeled drug, and rinsing the cells three times with ice-cold 1X PBS. Total radioactivity originating from the substrates was measured by lysing the cells with 1N NaOH, neutralized with 2N HCL, and measuring total radioactivity by liquid scintillation counting using an LS 6500 Counter (Beckman). Drug uptake results were normalized to total protein content and

then to data obtained in cells carrying an empty vector plasmid, which was set to 100%. The influence of inhibitors, including nilotinib (10 μM; 15-min pre-incubation) and rifampicin (10 μM; 15-min pre-incubation) on OATP1B1, OATP1B3, or OATP1B2 function was evaluated using EβG (Axxora LLC) as the prototypical substrate.

For the cellular vincristine uptake screening, human cervical carcinoma cells (HeLa) were transfected with transporters of interest, including hOAT1, hOAT3, hOAT4, hOATP1A2, hOATP3A1, hOATP4A1, hOATP5A1, hOATP1C1, hOATP1B1, hOATP1B3, hOATP2B1, hOCT1, hOCT2, hOCTN1, hOCTN2, hASBT, hNTCP, hNPT1, hMATE1, hOSTα/β, hPEPT1, hPEPT2, rOatp1a1, rOatp1a4, rOatp1a5, rOatp1b2, rNtcp, rAsbt, rOat1, and rOct1. Cells were transfected with expression plasmid or vector control lacking any insert using Lipofectin reagent (Invitrogen). Sixteen hours thereafter, cells were washed with transport media (OPTIMEM, Invitrogen) and treated with radiolabeled drug. At various time intervals, cells were washed three times with ice-cold PBS and then lysed with 1% sodium dodecyl sulfate. The cellular radioactivity was quantified by liquid scintillation counting as described above. Similar analyses were performed in HEK293 cells transfected with hOATP1A2, hOATP1C1, mMATE1, and hMATE1, with cells seeded in poly lysine pre-coated 12-well plates. For hOATP1A2 and hOATP1C1, transient transfections were performed with either an empty vector, OATP1A2, or OATP1C1 using Lipofectamine 2000 according to the manufacturers protocol (Invitrogen). At 48 h post transfection, uptake studies were performed with [^{3}H]estrone-3-sulfate (E-3-S) or vincristine in hOATP1A2-overexpressing cells and EβG or vincristine in hOATP1C1-overexpressing cells. For studies involving HEK293 cells transfected with MATE1, tetraethylammonium (TEA) was used as a positive control substrate in potassium-based buffers at pH 8.4 (instead of PBS), as described previously (13).

Gene expression analysis. Human DRG total RNA was purchased from Clontech Laboratories (Mountain View, CA) and was obtained post-mortem from 21 male/female white subjects (16 to 65 years old). RNA from cells was extracted using an Omega Extraction kit, according to manufacturer's protocol. Samples were analyzed using real-time quantitative PCR and Taqman Probes (Applied Biosystems). The relative gene expression was determined using the ΔΔCt method, and normalized to the mouse or human housekeeping gene, GAPDH. Amplified products were later visualized after separation of bands on a 2%

(wt/vol) agarose gel as described previously (14). OATP1B1, OATP1B3, and reduced folate carrier protein 1 (RFC1) gene expression from tumor samples of 314 pediatric patients with acute myeloid leukamia and 655 pediatric patients with acute lymphoblastic leukemia were obtained from cancer genomics data on the St. Jude Cloud (15). RFC1 was used as reference gene in tumor samples (16).

Antitumor efficacy evaluation. The influence of nilotinib on vincristine activity against cancer cells was performed on the ATCC TCP-1010 Leukemia Cell Line Panel (ie, Kasumi-1, HL-60, THP-1, K-562, RS4;11, Molt-4, and Ccrf-Cem). For *in vitro* cytotoxicity studies, stock solutions of nilotinib and vincristine were prepared in DMSO. The cell growth inhibitory potential of vincristine was evaluated with an MTT Cell Proliferation and Viability Assay following a 72-h continuous exposure. In brief, 50 µL containing 10,000 cells were plated in a 96-well format and exposed to a range of vincristine concentrations (up to 1 µM; at a 1:2 ratio) in the presence or absence of 1 uM nilotinib for 69 h at 37°C. The MTT dye was added 69 h after initiation and incubated at 37°C for an additional 3 h. The MTT dye is reduced by metabolically active and proliferating cells to form insoluble formazan crystals, which are solubilized by an acidified isopropyl alcohol solution, and the colorimetric output determined by using a multi-plate reader with 570 and 650 excitation and emission filters, respectively. The data were normalized to an untreated, proliferating condition at the 72-h time point, and set to 100%. A nonlinear sigmoidal curve fit was used to obtain IC_{50} values.

Tritium-labeled vincristine (ART 03310) was purchased from American Radiolabeled Chemicals. Prior to cellular accumulation studies, 1 million leukemic cells were transferred into 1.5 mL microcentrifuge tubes. In brief, the cells were spun down to remove growth medium and warm 1X PBS was added to rinse the cells of residual growth serum. The cells were then incubated for 5 min in a 500 µL solution containing 1 µM radiolabeled vincristine in the presence or absence of 0.1% DMSO or 1 µM nilotinib. After incubation, the cells were washed three times with cold 1X PBS to remove extracellular radioactivity. The cells were lysed in a solution containing 1 N NaOH for 4 h and neutralized with 2 N HCl. Total radioactivity was counted using liquid scintillation and normalized to protein concentrations (BCA protein kit).

Global untargeted metabolomics study. The plasma and DRG samples from wild-type mice and OATP1B2(-/-) mice were analyzed for structurally named and unknown molecules using a non-targeted semi-quantitative LC-MS/MS and gas chromatography/mass spectrometry platforms (Metabolon, Durham, NC). Raw data were extracted, peak-identified and QC processed using Metabolon's hardware and software. Compounds were identified by comparison to library entries of purified standards or recurrent unknown entities. Metabolon maintains a library based on authenticated standards that contains the retention time/index (RI), mass to charge ratio (m/z), and chromatographic data (including MS/MS spectral data) on all molecules present in the library. Peaks were quantified using area-under-the-curve, each biochemical in raw area counts was rescaled to set the median equal to 1, and missing values were imputed. Welch's two-sample *t*-test was used to identify biochemicals that differed significantly between experimental groups.

Statistical analysis. All data represent the mean and SEM before and/or after normalization to baseline values and are expressed as a percentage, unless stated otherwise. All experiments were performed using multiple replicates and were performed independently on at least two independent occasions. An unpaired two-sided Student's t test with Welch's correction was used for comparisons between two groups, and a one-way ANOVA with Dunnett's post hoc test was used for comparing more than two groups. Behavioral data were analyzed using two-way ANOVA with Tukey's or Bonferroni's post hoc test, repeated across time-points and groups. A $P < 0.05$ was used as the statistical cutoff across all analysis.

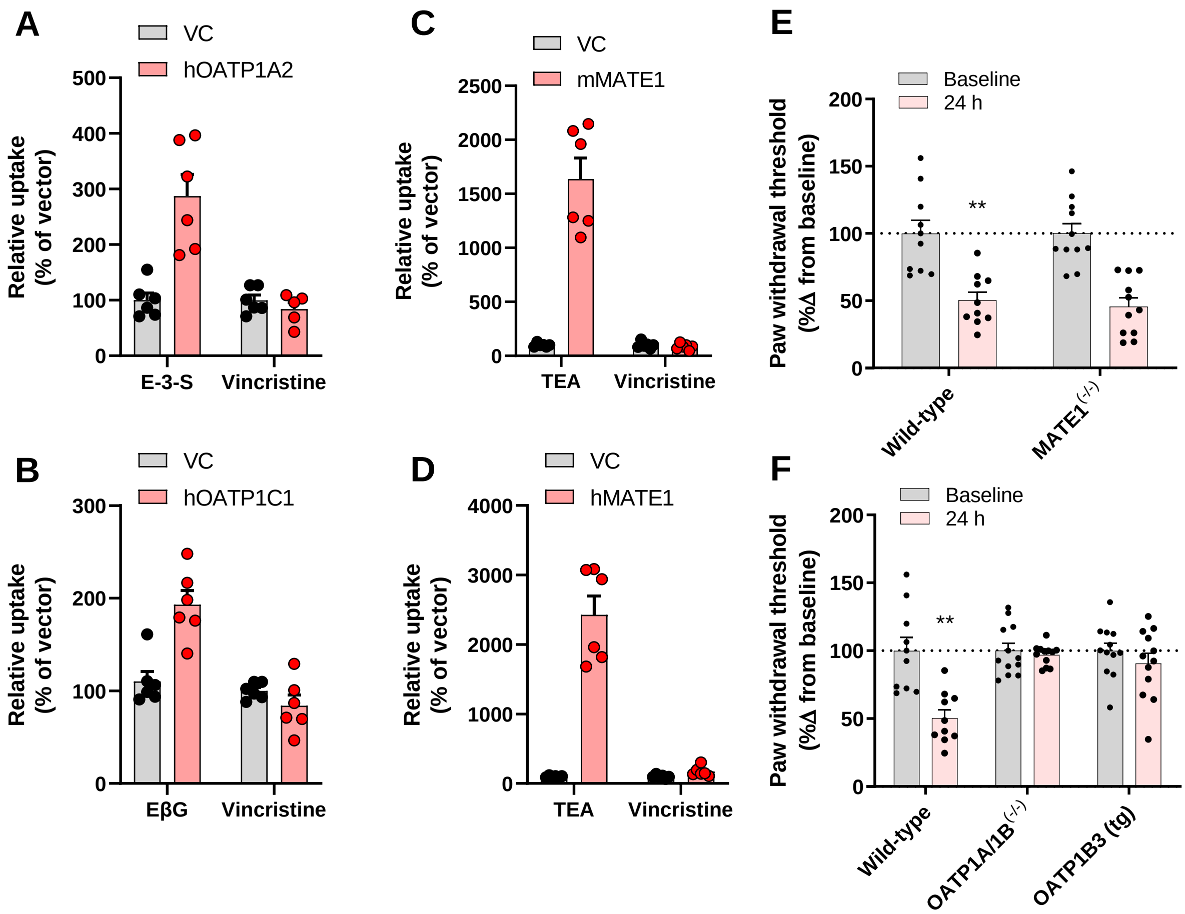

Figure S1. Validation of vincristine transport and toxicity in secondary *in vitro* and *in vivo* models. Transport of positive control substrates estrone-3-sulfate (E-3-S), tetraethylammonium (TEA), estradiol-17β-ᴅ-glucuronide (EβG), or vincristine in HEK293 cells engineered to overexpress human (h) OATP1A2 (A), hOATP1C1 (B), mouse (m) MATE1 (C) or hMATE1 (D). Each bar represents mean ± SD of two independent experiments (n = 6), with uptake data normalized to values observed in cells transfected with an empty vector control (VC). (E) and (F) Vincristine-induced mechanical withdrawal threshold reduction showed no significant differences between wild-type mice and MATE1-deficient mice (E), while deficiency of all OATP1A- and OATP1B-type transporters (OATP1A/1B KO) and OATP1A/1B-KO mice with transgenic (tg) overexpression of hOATP1B3 (F) were protected against this phenotype. All mice received a single 1 mg/kg dose of vincristine (n = 10-12 per group). Data presented represent the mean (bars) ± SEM (error bars), and statistical analyses were performed using one-way ANOVA with Dunnett's post hoc test: $^{**}P < 0.01$, compared to baseline values.

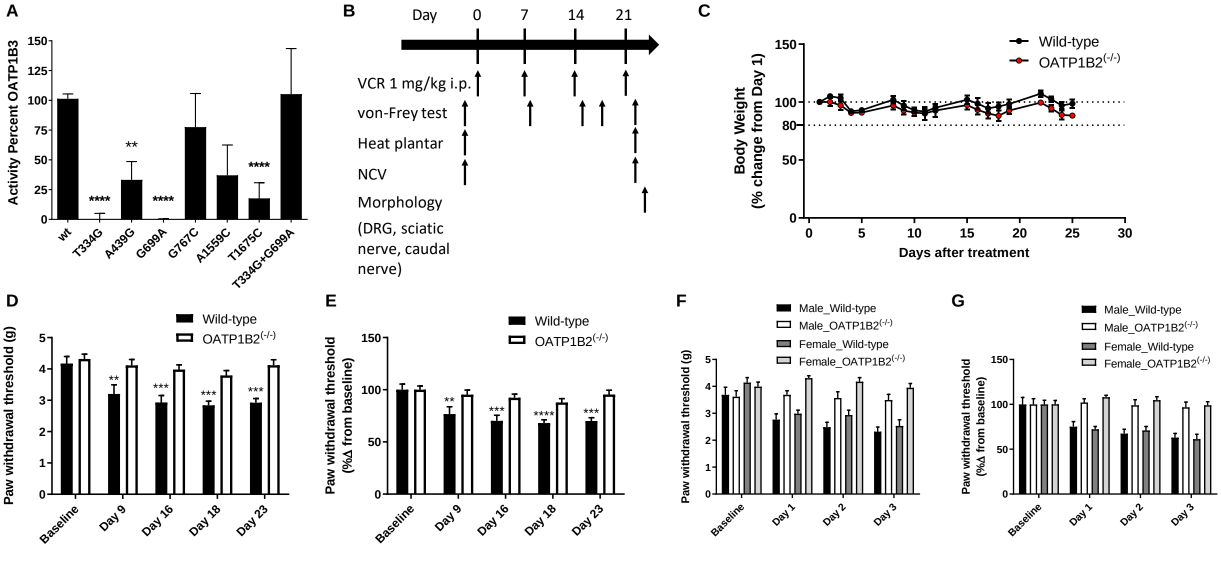

Figure S2. Role of OATP1B in vincristine-induced sensory peripheral neurotoxicity. (A) Single nucleotide polymorphisms (SNPs) activity percentage of OATP1B3 over-expressed cell after treated vincristine (n = 20 per group). Statistical analysis was performed using one-way ANOVA with Dunnett's post hoc test: ***P* < 0.01, *****P* < 0.0001, compared to wild-type mice. (B) Schematic overview of vincristine-induced sensory peripheral neurotoxicity: female wild-type mice and OATP1B2-deficient [OATP1B2(-/-)] mice received an i.p. injection of 1 mg/kg vincristine or the corresponding vehicle once a week (cumulative dose of 4 mg/kg). Von-Frey tests were performed on day -1, 9, 16, 18, and 23. Thermal hyperalgesia (Hargreaves test) and nerve conduction studies of the sciatic and caudal nerve were measured on days -1 and 23. Tissue samples for morphologic evaluation were obtained on day 24. (C) Vincristine-treated mice developed a slight weight loss over the course of the treatment in both wild-type and OATP1B2- deficient groups. (D) and (E) Mechanical allodynia changes in wild-type mice and OATP1B2(-/-) mice receiving 4 weekly 1 mg/kg doses of vincristine. Normalized mechanical allodynia (E) was expressed as a percentage change relative to baseline values (n = 6-8 per group). Statistical analysis was performed using a two-way ANOVA with Bonferroni's post hoc test: ***P* < 0.01, ****P* < 0.001, *****P* < 0.0001, compared with baseline values. (F) and (G) Vincristine- induced mechanical withdrawal threshold reduction showed no significant differences between male and female mice receiving a single 1 mg/kg dose of vincristine (n = 7-9 per group). Data presented represent the mean (bars or symbols) ± SEM (error bars).

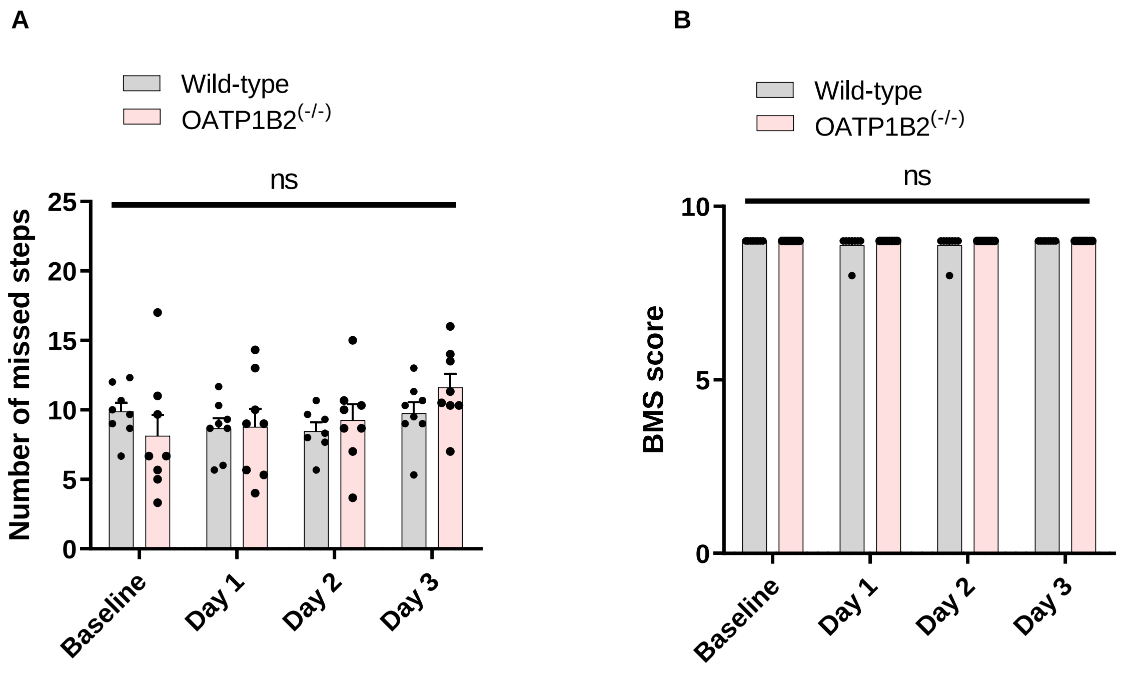

Figure S3. Vincristine does not affect gross motor function regardless of OATP1B2 genotype. (A) Gross motor function of wild-type mice or OATP1B2 deficient [OATP1B2(-/-)] mice at baseline and 1-, 2-, and 3-days post-administration of a single i.p. dose of vincristine (1 mg/kg; n = 8 per group) as assessed by an automated ladder test. (B) Open field evaluation of wild-type mice or OATP1B2(-/-) mice at baseline and 1-, 2-, and 3-days post-administration of a single i.p. dose of vincristine (1 mg/kg; n = 8 per group) using the mean Basso Mouse Scale for Locomotion (BMS) score. There were no pronounced differences observed in response to vincristine administration in either wild-type mice or OATP1B2(-/-) mice and both groups had an average score of 9, which represents normal locomotor behavior. ns denotes not statistically significant.

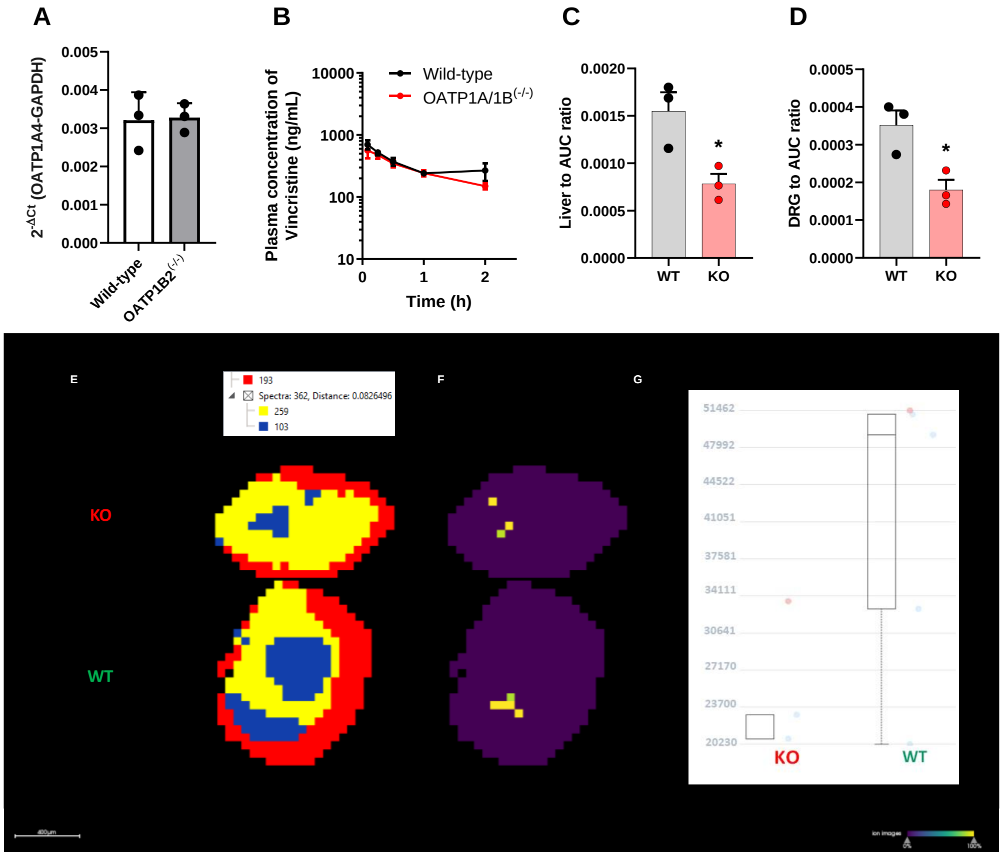

Figure S4. Role of OATP1B in vincristine tissue accumulation. (A) Gene expression of OATP1A4 in DRG specimens isolated from untreated wild-type mice or OATP1B2-deficient [OATP1B2(-/-)] mice (n = 3 per group). (B) Plasma concentration-time curves profile of vincristine (1 mg/kg) in wild-type mice or OATP1A/1B-deficient mice (n = 7-14 per group). Accumulation of vincristine in liver (C) and DRG (D), expressed as tissue to plasma AUC ratio of vincristine (1 mg/kg) in wild-type mice or OATP1B2(-/-) mice (n = 3 per group). Statistical analysis was performed using unpaired two-sided Student's *t* test with Welch's correction: **P* < 0.05, compared to wild-type values. All data presented represent the mean (bars or symbols) ± SEM (error bars). Representative MALDI mass spectrometry imaging of vincristine distribution in the DRG (L4 and L5) were obtained at 30 min and indicate fragment segmentation of the images (E) and distribution of vincristine in different regions of the DRG of wild-type mice and OATP1B2(-/-) mice. The signal corresponding to vincristine was detected at 825.46 *m/z*. (G) Intensity boxes indicate that higher average signals were detected in wild-type mice compared OATP1B2(-/-) mice.

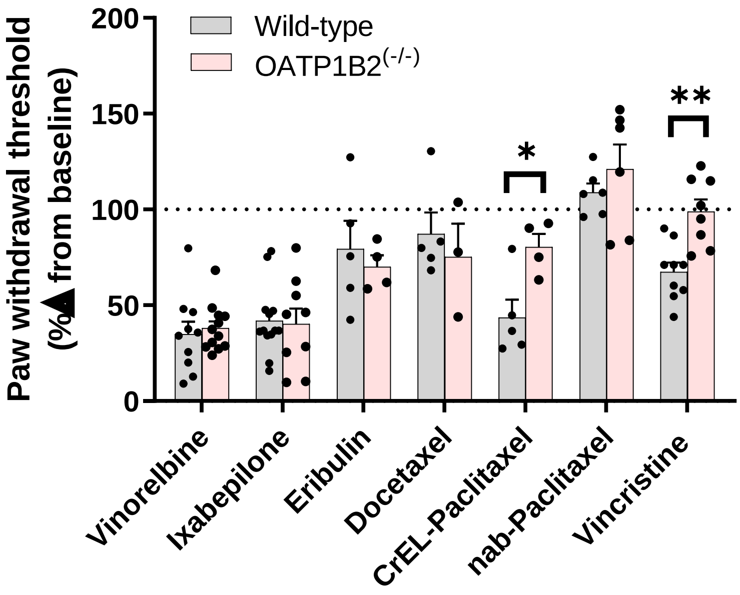

Figure S5. Role of OATP1B2 in microtubule targeting agents-induced peripheral neurotoxicity. Mechanical allodynia in wild-type mice and OATP1B2-deficient [OATP1B2(-/-)] mice 48 h after a single dose of vinorelbine (11 mg/kg, i.v.), ixabepilone (2 mg/kg, i.v.), eribulin (1.2 mg/kg, i.v.), docetaxel (10 mg/kg, i.v.), paclitaxel formulated in Cremophor EL-ethanol (CrEL-Paclitaxel; 10 mg/kg, i.v.), paclitaxel formulated as an albumin nanoparticle (nab-Paclitaxel; 10 mg/kg, i.v.), and vincristine (1 mg/kg, i.p.) (n = 5-13 per group). Data presented represent the mean (bars) ± SEM (error bars), and paw sensitivity is expressed as a percentage over baseline values. Statistical analysis was performed using an unpaired two-sided Student's *t* test with Welch's correction: **P* < 0.05, ***P* < 0.01, compared to wild-type values.

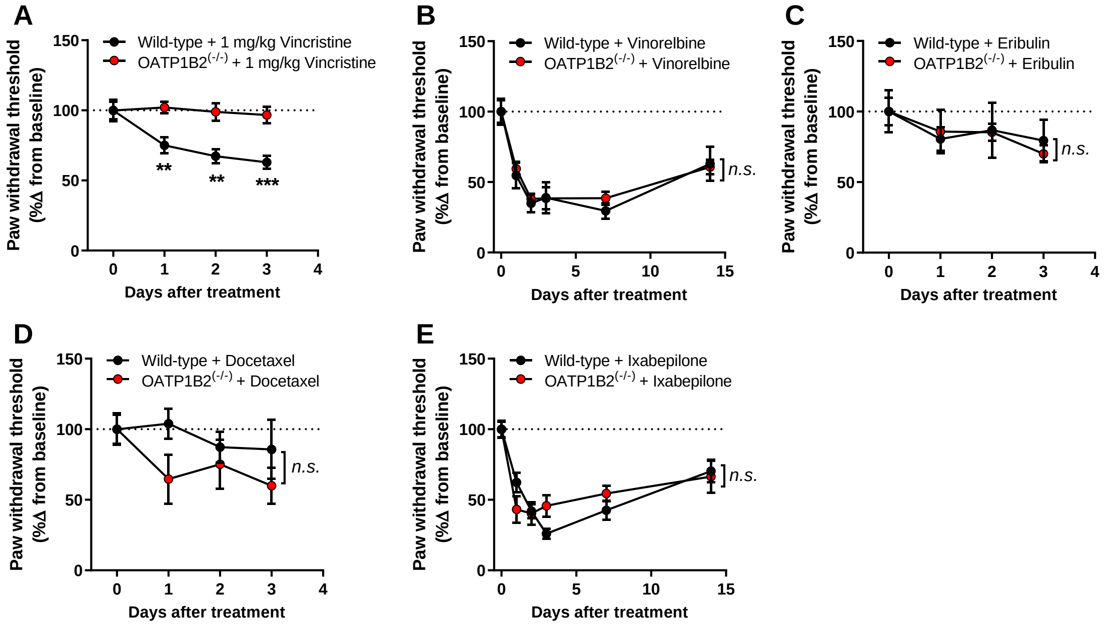

Figure S6. Time-course of microtubule targeting agents-induced mechanical allodynia. Mechanical allodynia was examined after single doses of vincristine (A), vinorelbine (B), eribulin (C), docetaxel (D), and ixabepilone (E) in wild-type mice or OATP1B2-deficient [OATP1B2(-/-)] mice (n = 5-13 per group). Paw sensitivity is expressed as a percentage over baseline values. Statistical analysis was performed using a two-way ANOVA with Bonferroni's post hoc test: ***P* < 0.01, ****P* < 0.001, compared to corresponding values in OATP1B2(-/-) mice values. All data presented represent the mean (symbols) ± SEM (error bars). n.s. denotes not statistically significant.

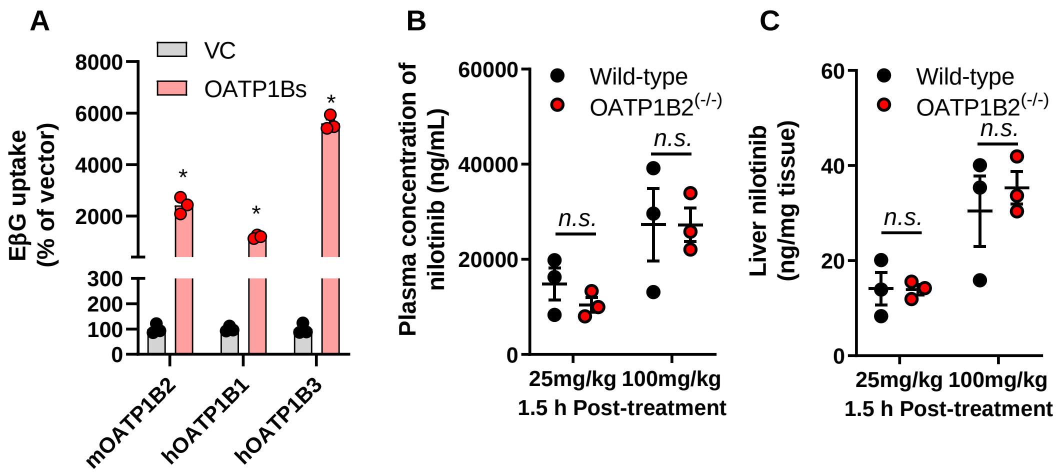

Figure S7. Identification of nilotinib as an inhibitor of OATP1B-type transporters. (A) Intracellular accumulation of EβG uptake into HEK293 cells overexpressing mouse (m) OATP1B2 or the human (h) OATP1B1 and OATP1B3 transporters. Relative uptake is expressed as percentage change compared with empty vector (VC) controls (n = 3 per group). Statistical analysis was performed using an unpaired two-sided Student's *t* test with Welch's correction: *$P < 0.05$, compared to VC. (B) Plasma concentration of nilotinib (25 mg/kg or 100 mg/kg, 1.5 h post-treatment) in wild-type mice or OATP1B2-deficient [OATP1B2(-/-)] mice (n = 3 per group). (C) Levels of nilotinib (25 mg/kg or 100 mg/kg, 1.5 h post-treatment) in livers of wild-type mice or OATP1B2(-/-) mice (n = 3 per group). All data presented represent the mean (bars or horizontal lines) ± SEM (error bars). n.s. denotes not statistically significant.

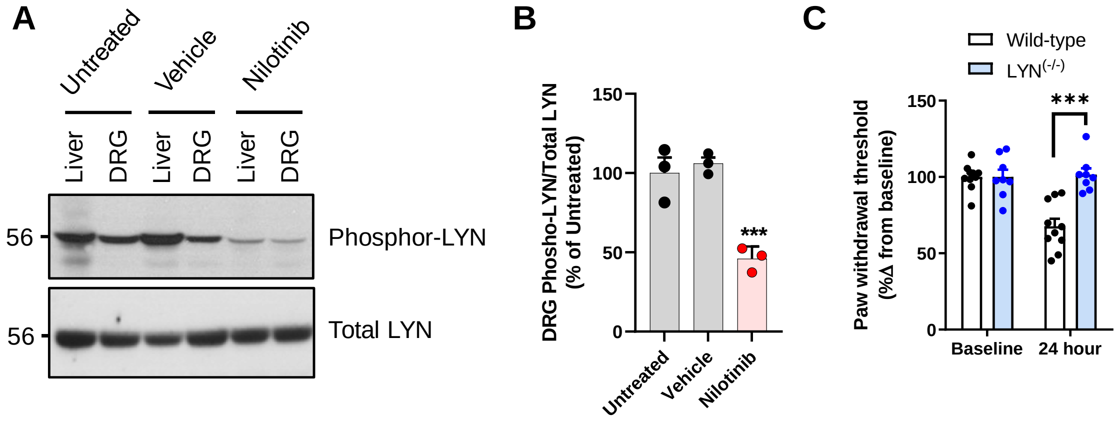

Figure S8. Influence of nilotinib administration on phosphorylation of LYN kinase in DRG. Levels of LYN kinase protein (LYN (#2796) and Phospho-LYN (#2731), Cell Signaling Technology) (A) and protein quantification (B) were evaluated in DRG samples from untreated, vehicle treated, or nilotinib treated (100 mg/kg; p.o.) wild-type mice, using specific antibodies for phospho-LYN and total LYN (n = 3 per group). (C) Mechanical allodynia in wild-type mice and LYN-deficient [LYN(-/-)] mice 24 h after a single dose of vincristine (1 mg/kg, i.p.) (n = 8-10 per group). Data presented represent the mean (bars) ± SEM (error bars), and paw sensitivity is expressed as a percentage over baseline values. Statistical analysis was performed using an unpaired two-sided Student's *t* test with Welch's correction: ****P* < 0.001.

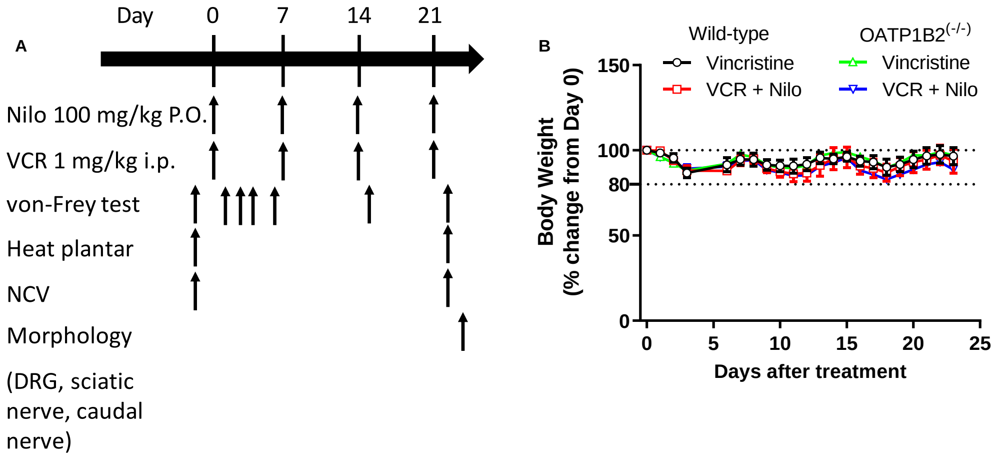

Figure S9. Effect of nilotinib-vincristine combination treatment on weight loss in mice. (A) Schematic overview of evaluation of the combinatorial treatment strategy and endpoint considerations over time. (B) Concurrent administration of nilotinib did not influence weight loss over time in wild-type mice and OATP1B2-deficient [OATP1B2(-/-)] mice. All mice received weekly i.p. injections of vincristine (1 mg/kg) preceded by vehicle (hydroxypropyl methylcellulose, HPMC) or nilotinib (100 mg/kg; p.o.)

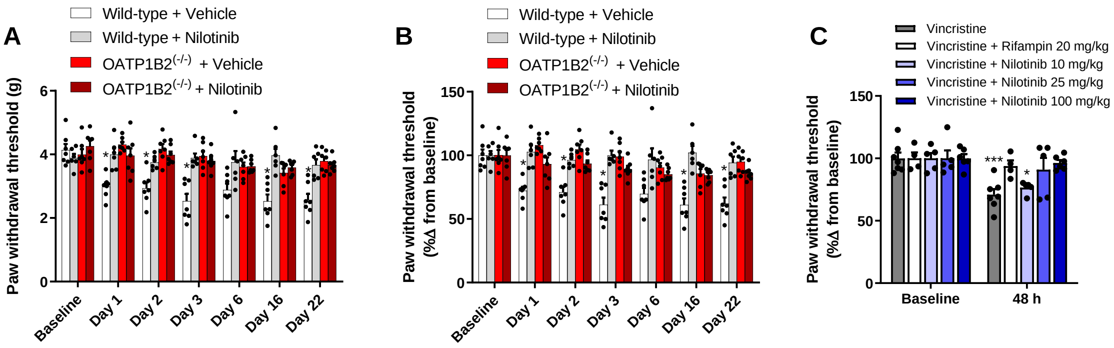

Figure S10. Inhibition of OATP1B2 protects against mechanical allodynia changes in mice receiving vincristine. (A) Absolute mechanical allodynia reading in wild-type mice or OATP1B2-deficient [OATP1B2(-/-)] mice receiving weekly i.p. injections of vincristine (1 mg/kg) and were pre-treated with vehicle alone (hydroxypropyl methylcellulose, HPMC) or nilotinib (100 mg/kg; p.o) 30-min prior to each vincristine dose. (B) Normalized mechanical allodynia readings, expressed as percentage change relative to baseline values (n = 6-7 per group). *P* < 0.05 compared to baseline values. (C) Influence of varying nilotinib doses (10, 25, or 100 mg/kg) and rifampin (20 mg/kg) on vincristine-induced mechanical allodynia in wild-type mice receiving single i.p. injections of vincristine (1 mg/kg) (n = 4-7 per group). Data presented represent the mean (bars) ± SEM (error bars). Statistical analysis was performed using a two-way ANOVA with Tukey's post hoc test: **P* < 0.05, ***P* < 0.01, compared with baseline values.

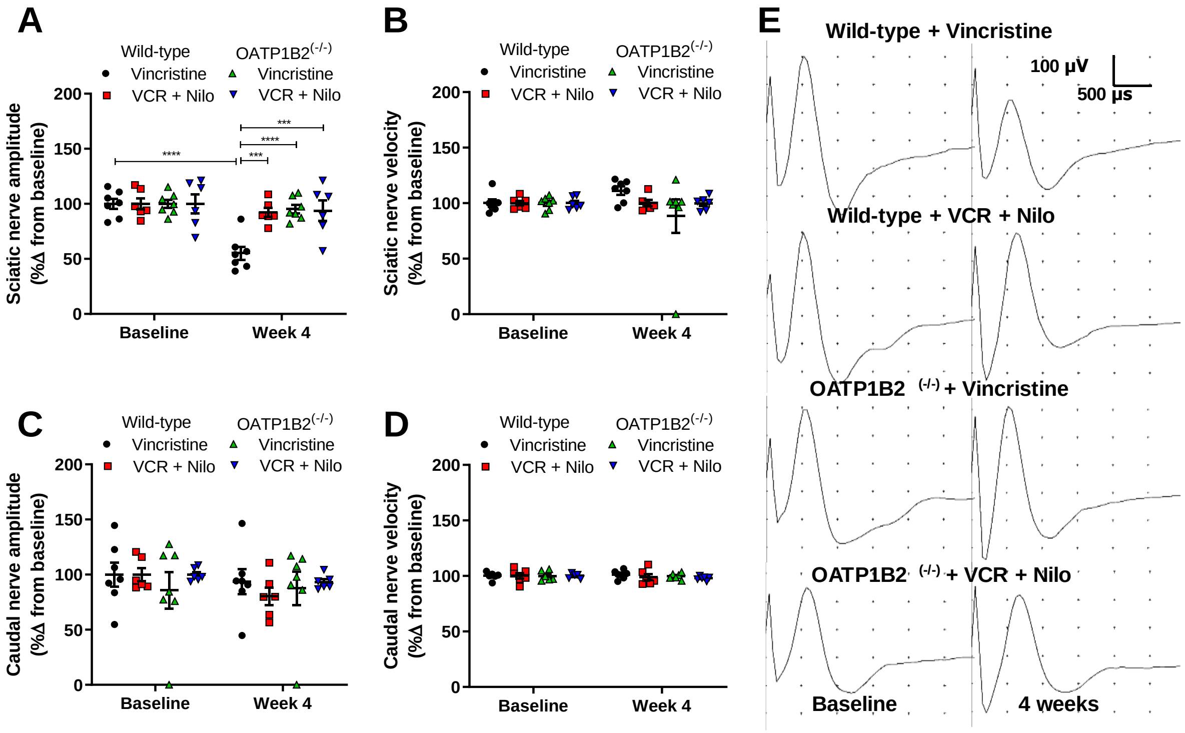

Figure S11. Effect of nilotinib on electrophysiological changes in mice receiving vincristine (VCR). Changes in sciatic nerve maximal action potential amplitudes (A), sciatic nerve conductance velocity (NCV) (B), caudal nerve maximal action potential amplitudes (C), and caudal nerve NCV (D) in wild-type mice or OATP1B2-deficient [OATP1B2(-/-)] mice receiving weekly i.p. injections of vincristine (1 mg/kg) . Mice were pre-treated with vehicle (hydroxypropyl methylcellulose) or nilotinib (Nilo; 100 mg/kg; p.o.) 30 min before every vincristine injection and representative electrophysiological readings are shown in panel (E). Data are expressed as percentage change relative to baseline values (n = 6-8 per group). Data presented represent the mean (horizontal lines) ± SEM (error bars). Statistical analysis was performed using a two-way ANOVA with Tukey's post hoc test: ***$P < 0.001$, ****$P < 0.0001$.

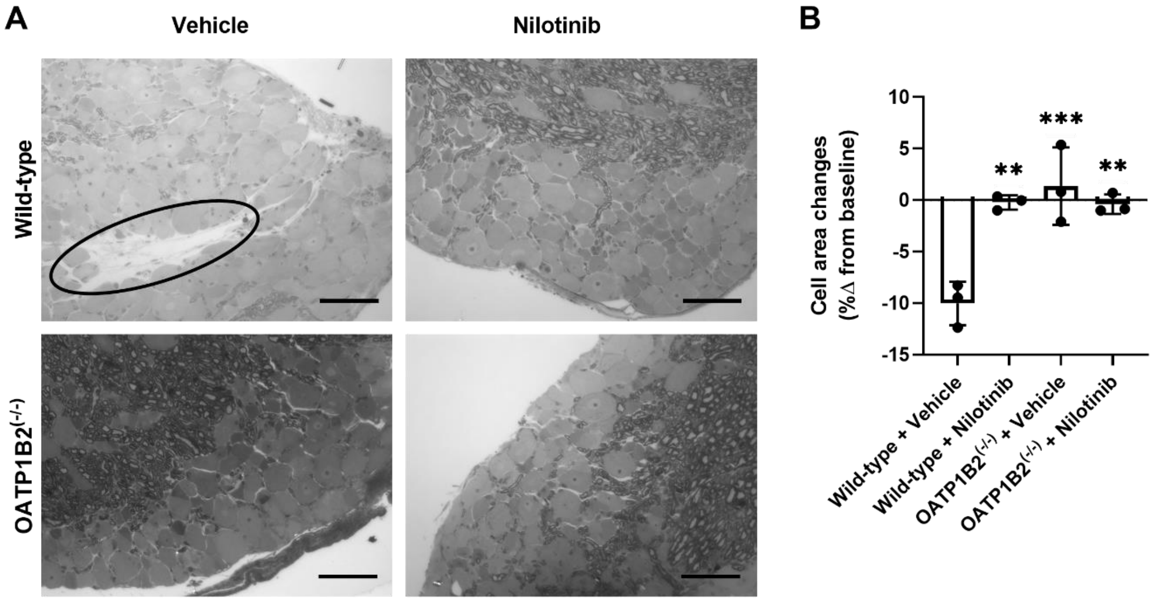

Figure S12. Protective effect of nilotinib on morphologic changes in the DRG of mice receiving vincristine. (A) Light microscopy analysis of the DRG of wild-type mice and OATP1B2-deficient [OATP1B2(-/-)] mice receiving weekly i.p. injections of vincristine at a dose of 1 mg/kg (cumulative dose 4 mg/kg). Mice were pre-treated with vehicle (hydroxypropyl methylcellulose) or nilotinib (100 mg/kg; p.o.) 30 min before every vincristine injection. After 4 weeks, vincristine-induced sensory neuron loss (circle) was evident in wild-type mice. Nilotinib pre-treatment and OATP1B2-deficiency protected against these morphologic changes. Magnification = 20×. Scale bar = 50 µm. (B) Vincristine-induced reduction in cell area in DRG specimens and prevention bynilotinib pre-treatment or OATP1B2 deficiency (n = 3 per group). Data presented represent the mean (bars) ± SEM (error bars). Statistical analysis was performed using one-way ANOVA with Dunnett's post hoc test: *$P < 0.05$.

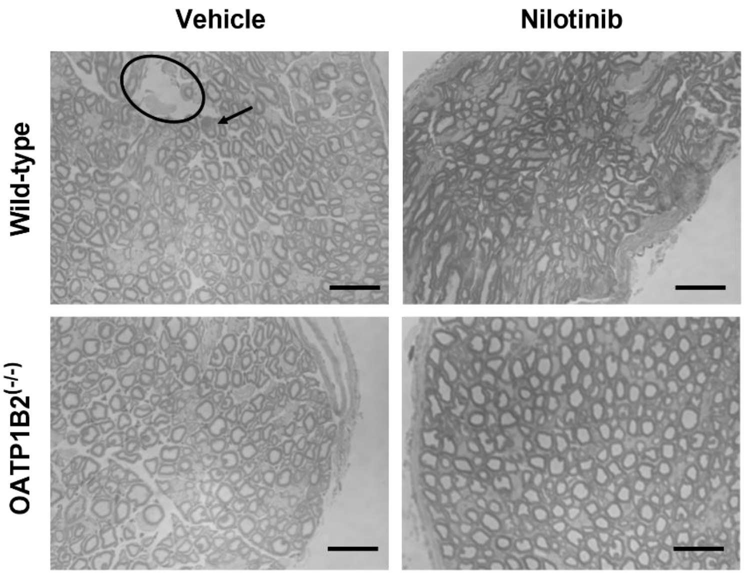

Figure S13. Protective effect of nilotinib on morphologic changes of the caudal nerve in mice receiving vincristine. Light microscopy analysis of the caudal nerve of wild-type mice and OATP1B2-deficient [OATP1B2(-/-)] mice receiving weekly i.p. injections of vincristine at a dose of 1 mg/kg (cumulative dose 4 mg/kg). Mice were pre-treated with vehicle (hydroxypropyl methylcellulose) or nilotinib (100 mg/kg; p.o.) 30 min before every vincristine injection. After 4 weeks, vincristine-induced axonopathy (arrow) and fiber loss (circle) were evident in wild-type mice, and nilotinib pre-treatment or OATP1B2 deficiency protected against these morphologic changes. Magnification = 63×. Scale bar = 20 μm.

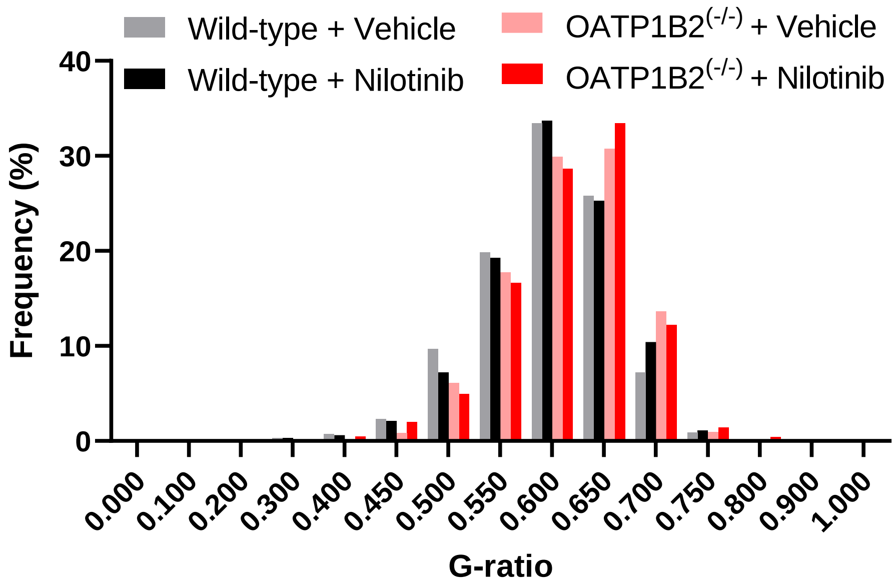

Figure S14. Protective effect of nilotinib on morphologic changes of the sciatic nerve in mice receiving vincristine. G-ratio (estimated by dividing the axon diameter by the myelinated fiber diameter) was calculated as a measure of myelination and axonal integrity of sciatic nerves. Vincristine induced a reduction in the frequency of the largest fibers (G-ratio = 0.7), and an increasing of the frequency of the smallest fibers (G-ratio = 0.5) in wild-type mice receiving weekly i.p. injections of vincristine at a dose of 1 mg/kg (cumulative dose 4 mg/kg), and pre-treatment of nilotinib or OATP1B2 deficiency [OATP1B2(-/-)] protected against these morphologic changes (n = 3 per group). Data presented represent the mean.

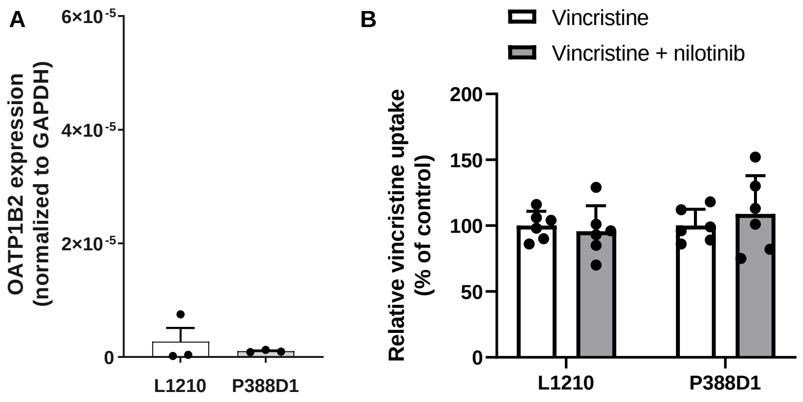

Figure S15. Nilotinib does not influence vincristine uptake into L1210 and P388D1 murine leukemia cells. (A) Gene expression of the OATP1B2 gene in L1210 and P388D1 murine leukemia cells (n = 3 per group). (B) Uptake of vincristine (1 µM, 30 min incubation) in L1210 and P388D1 murine leukemia cells in the presence or absence of nilotinib (10 µM) (n = 6 per group). Uptake data are normalized to total protein levels. All data presented represent the mean (bars) ± SEM (error bars).

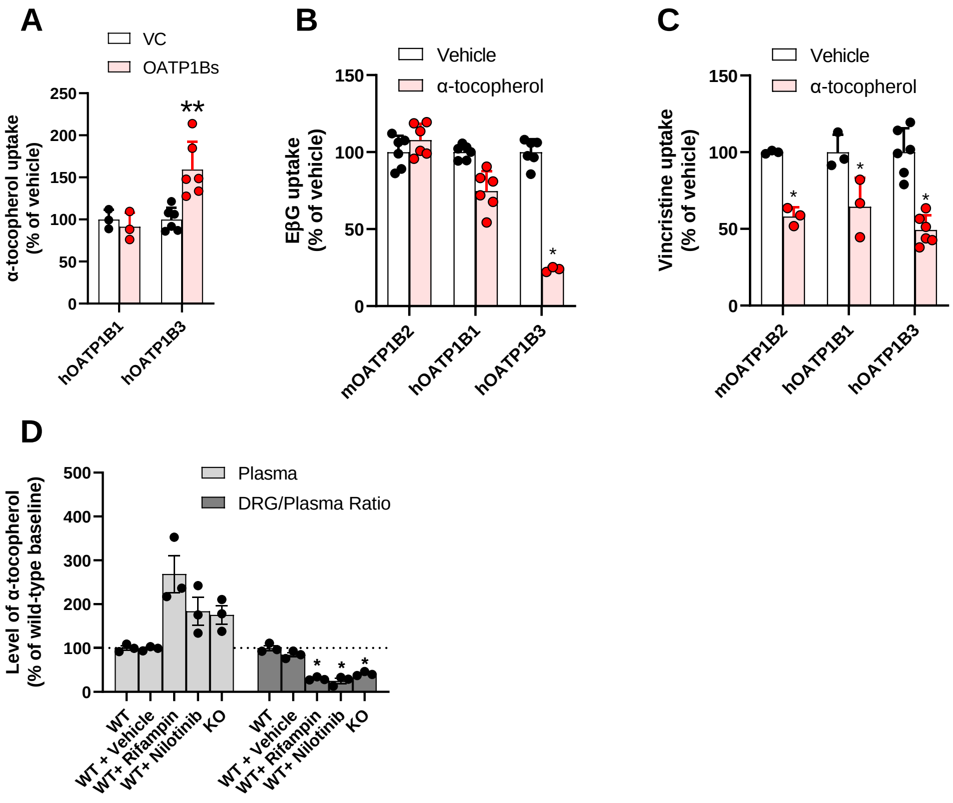

Figure S16. Identification of α-tocopherol as an endogenous neuronal biomarker of OATP1B-type transport. (A) Intracellular accumulation of α-tocopherol into HEK293 cells overexpressing human (h) OATP1B1 or OATP1B3. Relative uptake is expressed as percentage change compared with empty vector controls (VC) (n = 3-6 per group). Statistical analysis was performed using an unpaired two-sided Student's *t* test with Welch's correction: ***P* < 0.01, compared to vector control. α-tocopherol mediated inhibition of estradiol-17β-ᴅ-glucuronide (EβG) (B) and vincristine (C) uptake in HEK293 cells transfected with an empty vector (VC), mouse OATP1B2, OATP1B1, or OATP1B3. Uptake is expressed as percentage change compared with VC (n = 3-6 per group). Statistical analysis was performed using an unpaired two-sided Student's *t* test with Welch's correction: **P* < 0.05, compared with vehicle values. (D) Relative plasma level and DRG-to-plasma concentration ratio of α-tocopherol in wild-type mice or OATP1B2-deficient [OATP1B2(-/-)] mice 2 h after the administration of vehicle control (hydroxypropyl methylcellulose), rifampin (20 mg/kg; i.p.) or nilotinib (100 mg/kg; p.o.). Data are normalized to baseline levels in wild-type mice (n = 3 per group) and represent the mean (bars) ± SEM (error bars). Statistical analysis was performed using one-way ANOVA with Dunnett's post hoc test: **P* < 0.05.

Table S1. Pharmacokinetic parameters of vincristine in mice.

Genotype	Sex	N	C_{max} (ng/mL)[a]	$AUC_{0\text{-}4h}$ (ng×h/mL)[a]
Wild-type	Male	4	1190 ± 113	1060 ± 36.2
OATP1B2(-/-)	Male	4	1130 ± 148	1290 ± 49.8

[a] Data represent mean ± SEM.

Abbreviations: C_{max}, peak plasma concentration; $AUC_{0\text{-}4h}$, area under the plasma concentration-time curve between time zero and 4 hours; N, number of mice per group.

Table S2. Summary of drugs, formulations, treatment schedules, doses and test time points of MTAs-induced mechanical allodynia by Von Frey test.

Drugs	Formulation	Dose	Dose route	Time points (days post-treatment)
CrEL-Paclitaxel	Cremophor EL/ethanol (1:1, v/v), diluted with saline	10 mg/kg	i.v.	2
nab-Paclitaxel	Albumin-bound nanoparticle	10 mg/kg	i.v.	2
Vincristine	Saline	1 mg/kg	i.p.	1, 2, 3
Vinorelbine	Saline	11 mg/kg	i.v.	1, 2, 3, 7, 14
Eribulin	DMSO, diluted with saline	1.2 mg/kg	i.v.	1, 2, 3
Docetaxel	Polysorbate 80/ethanol (1:1, v/v), diluted with saline	10 mg/kg	i.v.	1, 2, 3
Ixabepilone	Cremophor EL/ethanol (1:1, v/v), diluted with PBS	2 mg/kg	i.v.	1, 2, 3, 7, 14
Vincristine	Saline	1 mg/kg once weekly, cumulative dose 4 mg/kg	i.p.	9, 16, 18, 23

Table S3. Influence of nilotinib on the pharmacokinetic parameters of vincristine in mice.

Genotype	Treatment group	N	C_{max} (ng/mL)[a]	AUC_{0-4h} (ng×h/mL)[a]
Wild-type	Vehicle	7	399 ± 30.9	452 ± 24.6
OAPT1B2(-/-)	Vehicle	8	509 ± 32.1	536 ± 25.3
Wild-type	Nilotinib	9	337 ± 16.9	426 ± 34.3
OATP1B2(-/-)	Nilotinib	9	443 ± 24.1	487 ± 7.37

[a] Data represent mean ± SEM.

Abbreviations: C_{max}, peak plasma concentration; AUC_{0-4h}, area under the plasma concentration-time curve between time zero and 4 hours; N, number of mice per group.

Table S4. Expression of OATP1B1 and OATP1B3 genes in human leukemia cells**.**

Sample Name	OATP1B1 (CT value)	OATP1B3 (CT value)	GAPDH (CT value)
**CCRF**	37.4	Undetermined	19.7
**CCRF**	37.8	41.4	19.7
**CCRF**	Undetermined	Undetermined	19.5
**MOLT4**	37.6	Undetermined	19.1
**MOLT4**	Undetermined	Undetermined	19.0
**MOLT4**	Undetermined	Undetermined	19.1
**RS411**	38.1	Undetermined	18.9
**RS411**	39.2	Undetermined	18.9
**RS411**	Undetermined	Undetermined	19.0
**HL60**	Undetermined	Undetermined	17.6
**HL60**	37.9	Undetermined	17.6
**HL60**	38.7	Undetermined	17.5
**K562**	37.8	Undetermined	19.2
**K562**	39.2	Undetermined	19.2
**K562**	Undetermined	Undetermined	19.2
**THP1**	Undetermined	Undetermined	19.5
**THP1**	Undetermined	Undetermined	19.6
**THP1**	Undetermined	Undetermined	19.9
**hOATP1B1 cell**	22.6	Undetermined	19.0
**hOATP1B1 cell**	22.6	Undetermined	19.0
**hOATP1B1 cell**	22.3	Undetermined	19.1
**hOATP1B3 cell**	Undetermined	20.1	18.8
**hOATP1B3 cell**	Undetermined	20.0	18.8
**hOATP1B3 cell**	Undetermined	20.0	18.8

Table S5. Genetic variants in OATP1B3 associated with vincristine induced peripheral neuropathy in children with acute lymphoblastic leukemia.*

Variant ID (chr:position:Ref:Alt_EffectAllele; hg38)	Estimate of effect allele	Unadjusted *P*-value	Adjusted *P*-value	Allele frequency of effect allele	rsID	Variant function
chr12:20836048:G:A_A	.642	.00300	.698	.807	rs1072364	intronic
chr12:20905218:G:T_T	-1.12	.0260	.990	.0430	rs7957904	intronic
chr12:20908187:TTAAA:T_T	-1.12	.0260	.990	.0430	rs112836316	intronic
chr12:20909124:G:C_C	.506	.0300	.994	.816	rs10770758	intronic
chr12:20826554:CT:C_C	.797	.0390	.998	.0490	rs397954123	intronic
chr12:20905668:G:T_T	-.956	.0420	1.00	.0440	rs58213259	intronic

*A total of 669 variants with minor allele frequency >3% within 10k bps flanking of the OATP1B3 gene *SLCO1B3* were tested for association between genotypes and vincristine-induced peripheral neurotoxicity in the cohort of 321 pediatric patients with acute lymphoblastic leukemia (17). Shown are single-nucleotide polymorphisms (SNPs) with $P < 0.05$ without adjusting for multiple testing. Permutation teste were used to generate adjusted *P*-values for multiple testing.

Table S6. Differentially circulating endogenous metabolites in untreated plasma of wild-type mice or OATP1B2-deficient (KO) mice. Names in red indicate previously identified putative OATP1B-type transport biomarkers (18). Positive fold changes indicate higher plasma levels in OATP1B2-deficient mice. Statistical analysis was performed using Welch's two-sample *t*-test.

Biochemical Name	Fold of Change KO/WT	*P*-value KO/WT	Mean value KO	Mean value WT	Sub Pathway
**chenodeoxycholic acid 24-glucuronide**	**97.9**	.000	1.15	.0120	Primary Bile Acid Metabolism
**alpha-muricholate**	**45.2**	.000	2.71	.0600	Primary Bile Acid Metabolism
**ursocholate**	**33.8**	.000	5.10	.151	Secondary Bile Acid Metabolism
4-ethylphenylsulfate	**17.2**	.000	4.08	.238	Benzoate Metabolism
**beta-muricholate**	**15.2**	.000	2.84	.187	Primary Bile Acid Metabolism
**hyodeoxycholate**	**14.7**	.000	3.94	.269	Secondary Bile Acid Metabolism
**cholate**	**13.7**	.000	3.61	.263	Primary Bile Acid Metabolism
6-beta-hydroxylithocholate	**13.1**	.000	1.16	.0890	Secondary Bile Acid Metabolism
octadecenedioylcarnitine (C18:1-DC)*	**12.7**	.000	2.35	.185	Fatty Acid Metabolism (Acyl Carnitine, Dicarboxylate)
p-cresol glucuronide*	**11.8**	.000	5.92	.503	Tyrosine Metabolism
**deoxycholate**	**10.0**	.000	2.50	.249	Secondary Bile Acid Metabolism
p-cresol sulfate	**9.29**	.000	4.66	.501	Benzoate Metabolism
1-oleoyl-GPG (18:1)*	**8.19**	.000	1.80	.219	Lysophospholipid
dodecanedioate (C12-DC)	**8.12**	.000	2.97	.366	Fatty Acid, Dicarboxylate
bilirubin degradation product, C17H20N2O5 (1)**	**7.14**	.000	2.40	.336	Partially Characterized Molecules
bilirubin degradation product, C16H18N2O5 (1)**	**6.67**	.000	1.23	.185	Partially Characterized Molecules
branched chain 14:0 dicarboxylic acid**	**6.55**	.000	1.80	.275	Fatty Acid, Dicarboxylate
chenodeoxycholic acid sulfate (1)	**6.41**	.00600	1.02	.160	Primary Bile Acid Metabolism
7-ketodeoxycholate	**6.23**	.00100	2.26	.362	Secondary Bile Acid Metabolism
bilirubin degradation product, C16H18N2O5 (3)**	**6.13**	.000	1.96	.319	Partially Characterized Molecules
**bilirubin (E,E)***	**6.12**	.000	1.17	.191	Hemoglobin and Porphyrin Metabolism
**hyocholate**	**5.64**	.000	1.41	.251	Secondary Bile Acid Metabolism
bilirubin degradation product, C17H18N2O4 (2)**	**5.59**	.000	2.14	.382	Partially Characterized Molecules
1-palmitoyl-GPG (16:0)*	**5.40**	.000	2.00	.371	Lysophospholipid
eicosenedioate (C20:1-DC)*	**5.36**	.000	1.80	.335	Fatty Acid, Dicarboxylate
**bilirubin (Z,Z)**	**5.25**	.00100	1.48	.282	Hemoglobin and Porphyrin Metabolism
bilirubin degradation product, C17H18N2O4 (1)**	**5.05**	.000	2.10	.416	Partially Characterized Molecules
3-hydroxydodecanedioate*	**4.32**	.000	1.64	.380	Fatty Acid, Dicarboxylate
2-amino-p-cresol sulfate	**4.28**	.00100	1.86	.435	Benzoate Metabolism
**tetradecanedioate (C14-DC)**	**4.27**	.000	1.96	.459	Fatty Acid, Dicarboxylate
1-stearoyl-GPG (18:0)	**4.16**	.000	1.76	.423	Lysophospholipid
biliverdin	**4.09**	.000	1.84	.450	Hemoglobin and Porphyrin Metabolism

**ursodeoxycholate**	**4.07**	.00700	2.16	.530	Secondary Bile Acid Metabolism
dodecenedioate (C12:1-DC)*	**3.94**	.000	2.14	.543	Fatty Acid, Dicarboxylate
1-linoleoyl-GPG (18:2)*	**3.71**	.000	1.96	.529	Lysophospholipid
bilirubin degradation product, C17H18N2O4 (3)**	**3.65**	.000	1.81	.496	Partially Characterized Molecules
bilirubin degradation product, C16H18N2O5 (2)**	**3.62**	.000	1.75	.482	Partially Characterized Molecules
hexadecenedioate (C16:1-DC)*	**3.61**	.000	1.62	.448	Fatty Acid, Dicarboxylate
**chenodeoxycholate**	**3.57**	.000	2.09	.585	Primary Bile Acid Metabolism
bilirubin degradation product, C17H20N2O5 (2)**	**3.53**	.000	2.00	.567	Partially Characterized Molecules
octadecadienedioate (C18:2-DC)*	**3.48**	.000	1.88	.540	Fatty Acid, Dicarboxylate
N,N,N-trimethyl-5-aminovalerate	**3.45**	.000	2.06	.597	Lysine Metabolism
**taurodeoxycholate**	**3.45**	.00100	1.82	.528	Secondary Bile Acid Metabolism
enterolactone sulfate	**3.42**	.000	1.63	.478	Food Component/Plant
glucuronate	**3.17**	.000	1.59	.500	Aminosugar Metabolism
2-hydroxy-3-methylvalerate	**3.15**	.00400	2.33	.738	Leucine, Isoleucine and Valine Metabolism
bilirubin degradation product, C16H18N2O5 (4)**	**3.06**	.000	1.58	.514	Partially Characterized Molecules
undecanedioate (C11-DC)	**3.00**	.000	1.96	.654	Fatty Acid, Dicarboxylate
octadecenedioate (C18:1-DC)	**2.66**	.000	1.45	.544	Fatty Acid, Dicarboxylate
equol sulfate	**2.58**	.000	1.62	.626	Food Component/Plant
isohyodeoxycholate	**2.46**	.00900	0.940	.382	Secondary Bile Acid Metabolism
phenylpropionylglycine	**2.41**	.00600	1.58	.656	Benzoate Metabolism
1-linoleoyl-GPA (18:2)*	**2.36**	.00300	1.41	.595	Lysophospholipid
alpha-hydroxyisocaproate	**2.29**	.0130	1.80	.787	Leucine, Isoleucine and Valine Metabolism
taurolithocholate 3-sulfate	**2.25**	.0160	0.960	.425	Secondary Bile Acid Metabolism
tauroursodeoxycholic acid sulfate (2)	**2.18**	.0130	1.12	.513	Secondary Bile Acid Metabolism
1-(1-enyl-oleoyl)-GPE (P-18:1)*	**2.16**	.000	1.45	.672	Lysoplasmalogen
pregnenediol disulfate (C21H34O8S2)*	**2.14**	.0300	1.16	.540	Pregnenolone Steroids
4-acetamidobenzoate	**2.14**	.00400	1.46	.685	Chemical
1-linolenoyl-GPC (18:3)*	**2.13**	.000	1.41	.661	Lysophospholipid
glucuronide of C10H18O2 (11)*	**2.12**	.00100	1.57	.737	Partially Characterized Molecules
glucuronide of C14H22O4 (1)*	**2.12**	.000	1.47	.690	Partially Characterized Molecules
**hexadecanedioate (C16-DC)**	**2.10**	.00100	1.57	.749	Fatty Acid, Dicarboxylate
tetradecadienedioate (C14:2-DC)*	**2.09**	.00600	1.58	.756	Fatty Acid, Dicarboxylate
inosine	**2.07**	.0320	1.49	.719	Purine Metabolism, (Hypo)Xanthine/Inosine containing
1-oleoyl-GPE (18:1)	**2.05**	.000	1.30	.634	Lysophospholipid
α-tocopherol	**2.03**	.000	1.44	.707	Tocopherol Metabolism
1,2-dipalmitoyl-GPE (16:0/16:0)*	**2.02**	.00100	1.34	.664	Phosphatidylethanolamine (PE)
1-oleoyl-2-arachidonoyl-GPI (18:1/20:4)*	**2.01**	.0120	1.37	.681	Phosphatidylinositol (PI)
cinnamoylglycine	**1.99**	.0240	1.54	.776	Food Component/Plant

1-palmitoleoyl-GPC (16:1)*	**1.97**	.000	1.31	.666	Lysophospholipid
2-palmitoleoyl-GPC (16:1)*	**1.95**	.00200	1.36	.696	Lysophospholipid
1-linoleoyl-GPE (18:2)*	**1.94**	.00400	1.42	.736	Lysophospholipid
4-ethylcatechol sulfate	**1.93**	.00100	1.56	.807	Benzoate Metabolism
arachidonoylcarnitine (C20:4)	**1.92**	.000	1.53	.796	Fatty Acid Metabolism (Acyl Carnitine, Polyunsaturated)
linolenoylcarnitine (C18:3)*	**1.91**	.000	1.51	.794	Fatty Acid Metabolism (Acyl Carnitine, Polyunsaturated)
equol glucuronide	**1.90**	.00600	1.35	.709	Food Component/Plant
phenol glucuronide	**1.87**	.0160	1.28	.684	Tyrosine Metabolism
1-oleoyl-GPI (18:1)	**1.87**	.00200	1.36	.729	Lysophospholipid
1-palmitoleoyl-2-linolenoyl-GPC (16:1/18:3)*	**1.86**	.00200	1.46	.784	Phosphatidylcholine (PC)
5-methyl-2'-deoxycytidine	**1.83**	.00200	1.56	.852	Pyrimidine Metabolism, Cytidine containing
4-hydroxycinnamate sulfate	**1.81**	.00900	1.28	.707	Tyrosine Metabolism
1-arachidonoyl-GPA (20:4)	**1.80**	.00800	1.17	.652	Lysophospholipid
1-(1-enyl-palmitoyl)-GPE (P-16:0)*	**1.79**	.000	1.30	.725	Lysoplasmalogen
1-oleoyl-GPC (18:1)	**1.78**	.000	1.30	.732	Lysophospholipid
2,8-quinolinediol sulfate	**1.78**	.0280	1.68	.946	Food Component/Plant
pentadecanoylcarnitine (C15)*	**1.77**	.000	1.28	.723	Fatty Acid Metabolism (Acyl Carnitine, Long Chain Saturated)
pyridoxamine	**1.77**	.000	1.42	.805	Vitamin B6 Metabolism
dihomo-linoleoylcarnitine (C20:2)*	**1.75**	.000	1.37	.785	Fatty Acid Metabolism (Acyl Carnitine, Polyunsaturated)
1-palmitoyl-GPE (16:0)	**1.73**	.000	1.33	.765	Lysophospholipid
butyrylglycine	**1.72**	.00700	1.28	.744	Fatty Acid Metabolism (also BCAA Metabolism)
dihomo-linolenoylcarnitine (C20:3n3 or 6)*	**1.72**	.000	1.37	.795	Fatty Acid Metabolism (Acyl Carnitine, Polyunsaturated)
1-(1-enyl-stearoyl)-GPE (P-18:0)*	**1.72**	.000	1.28	.747	Lysoplasmalogen
alpha-hydroxyisovalerate	**1.68**	.00300	1.43	.850	Leucine, Isoleucine and Valine Metabolism
heptadecanedioate (C17-DC)	**1.66**	.00100	1.36	.818	Fatty Acid, Dicarboxylate
1-linoleoyl-GPI (18:2)*	**1.65**	.0180	1.27	.765	Lysophospholipid
1-ribosyl-imidazoleacetate*	**1.64**	.0450	1.34	.818	Histidine Metabolism
palmitoleoylcarnitine (C16:1)*	**1.63**	.0210	1.54	.943	Fatty Acid Metabolism (Acyl Carnitine, Monounsaturated)
1-arachidonoyl-GPC (20:4n6)*	**1.63**	.000	1.27	.775	Lysophospholipid
octanoylcarnitine (C8)	**1.62**	.0330	1.47	.910	Fatty Acid Metabolism (Acyl Carnitine, Medium Chain)
linoleoylcarnitine (C18:2)*	**1.62**	.00400	1.36	.838	Fatty Acid Metabolism (Acyl Carnitine, Polyunsaturated)
1-linoleoyl-GPC (18:2)	**1.62**	.000	1.22	.752	Lysophospholipid
3-phenylpropionate (hydrocinnamate)	**1.62**	.0370	1.39	.855	Benzoate Metabolism
cinnamate	**1.60**	.0380	1.56	.975	Food Component/Plant
hexanoylglycine	**1.59**	.0480	1.40	.880	Fatty Acid Metabolism (Acyl Glycine)
margaroylcarnitine (C17)*	**1.59**	.00100	1.21	.764	Fatty Acid Metabolism (Acyl Carnitine, Long Chain Saturated)
1-(1-enyl-palmitoyl)-GPC (P-16:0)*	**1.59**	.000	1.26	.795	Lysoplasmalogen
**taurodeoxycholic acid 3-sulfate**	**1.58**	.0430	1.10	.694	Secondary Bile Acid Metabolism

isocaproylglycine	1.57	.0240	1.28	.815	Fatty Acid Metabolism (Acyl Glycine)
glycerophosphoethanolamine	1.57	.00200	1.31	.831	Phospholipid Metabolism
oleoylcarnitine (C18:1)	1.56	.0200	1.40	.900	Fatty Acid Metabolism (Acyl Carnitine, Monounsaturated)
mannitol/sorbitol	1.55	.00800	1.24	.800	Fructose, Mannose and Galactose Metabolism
4-vinylphenol sulfate	1.55	.00400	1.32	.847	Benzoate Metabolism
hydroquinone sulfate	1.55	.0230	1.32	.849	Drug - Topical Agents
valerylglycine	1.53	.0260	1.28	.838	Fatty Acid Metabolism (Acyl Glycine)
heptanoyl glycine	1.52	.0470	1.32	.868	Fatty Acid Metabolism (Acyl Glycine)
1-linoleoyl-2-linolenoyl-GPC (18:2/18:3)*	1.51	.0320	1.40	.923	Phosphatidylcholine (PC)
indolin-2-one	1.51	.0130	1.20	.797	Food Component/Plant
stearamide (18:0)	1.49	.0100	1.19	.800	Fatty Acid, Amide
1-arachidonoyl-GPE (20:4n6)*	1.49	.0110	1.20	.803	Lysophospholipid
6-hydroxyindole sulfate	1.49	.0130	1.14	.762	Chemical
3-indoxyl sulfate	1.48	.0190	1.19	.804	Tryptophan Metabolism
1-linoleoyl-2-arachidonoyl-GPE (18:2/20:4)*	1.48	.0280	1.26	.854	Phosphatidylethanolamine (PE)
1-arachidonoyl-GPI (20:4)*	1.48	.0220	1.11	.754	Lysophospholipid
pyridoxal	1.47	.000	1.28	.868	Vitamin B6 Metabolism
phenylacetylglutamate	1.46	.0370	1.24	.850	Acetylated Peptides
eicosadienamide (20:2)*	1.46	.0150	1.27	.869	Fatty Acid, Amide
5-dodecenoylcarnitine (C12:1)	1.46	.0420	1.37	.936	Fatty Acid Metabolism (Acyl Carnitine, Monounsaturated)
1-palmitoyl-2-oleoyl-GPE (16:0/18:1)	1.46	.0400	1.24	.847	Phosphatidylethanolamine (PE)
1-(1-enyl-palmitoyl)-2-arachidonoyl-GPC (P-16:0/20:4)*	1.46	.00100	1.30	.891	Plasmalogen
margaramide (17:0)*	1.45	.00900	1.19	.826	Fatty Acid, Amide
sebacate (C10-DC)	1.44	.0230	1.20	.833	Fatty Acid, Dicarboxylate
nonadecanedioate (C19-DC)	1.44	.00500	1.30	.902	Fatty Acid, Dicarboxylate
eicosenamide (20:1)*	1.43	.0300	1.27	.885	Fatty Acid, Amide
6-oxolithocholate	1.43	.0390	0.970	.678	Secondary Bile Acid Metabolism
N-acetylkynurenine (2)	1.41	.00300	1.17	.827	Tryptophan Metabolism
ethyl beta-glucopyranoside	1.41	.0210	1.17	.826	Food Component/Plant
phenol sulfate	1.40	.0340	1.23	.879	Tyrosine Metabolism
beta-hydroxyisovalerate	1.40	.0050	1.17	.835	Leucine, Isoleucine and Valine Metabolism
isobutyrylcarnitine (C4)	1.40	.0150	1.15	.817	Leucine, Isoleucine and Valine Metabolism
N-acetylmethionine sulfoxide	1.40	.0450	1.31	.931	Methionine, Cysteine, SAM and Taurine Metabolism
palmitamide (16:0)	1.40	.00700	1.18	.840	Fatty Acid, Amide
nonadecenamide (19:1)*	1.39	.0270	1.27	.917	Fatty Acid, Amide
myristoylcarnitine (C14)	1.39	.0420	1.26	.904	Fatty Acid Metabolism (Acyl Carnitine, Long Chain Saturated)
tigloylglycine	1.38	.0210	1.22	.883	Leucine, Isoleucine and Valine Metabolism
**octadecanedioate (C18-DC)**	1.38	.00300	1.27	.916	Fatty Acid, Dicarboxylate

oleamide	1.38	.0130	1.22	.886	Fatty Acid, Amide
heptadecenamide (17:1)*	1.37	.0330	1.29	.939	Fatty Acid, Amide
linoleamide (18:2n6)	1.36	.0420	1.29	.945	Fatty Acid, Amide
arachidoylcarnitine (C20)*	1.36	.0110	1.21	.889	Fatty Acid Metabolism (Acyl Carnitine, Long Chain Saturated)
1-linoleoyl-2-arachidonoyl-GPC (18:2/20:4n6)*	1.36	.00700	1.15	.846	Phosphatidylcholine (PC)
1-lignoceroyl-GPC (24:0)	1.36	.00700	1.16	.847	Lysophospholipid
sphingomyelin (d18:2/14:0, d18:1/14:1)*	1.36	.0320	1.14	.839	Sphingomyelins
N4-acetylcytidine	1.36	.00300	1.12	.819	Pyrimidine Metabolism, Cytidine containing
N-acetylhomocitrulline	1.35	.00100	1.17	.861	Urea cycle; Arginine and Proline Metabolism
2,3-dihydroxy-5-methylthio-4-pentenoate (DMTPA)*	1.34	.0140	1.23	.915	Methionine, Cysteine, SAM and Taurine Metabolism
glycerophosphorylcholine (GPC)	1.34	.000	1.12	.836	Phospholipid Metabolism
1-oleoyl-2-docosahexaenoyl-GPC (18:1/22:6)*	1.34	.00100	1.17	.874	Phosphatidylcholine (PC)
1-stearoyl-GPE (18:0)	1.34	.00200	1.16	.867	Lysophospholipid
1-(1-enyl-palmitoyl)-2-linoleoyl-GPE (P-16:0/18:2)*	1.34	.0290	1.16	.862	Plasmalogen
1-(1-enyl-palmitoyl)-2-arachidonoyl-GPE (P-16:0/20:4)*	1.34	.00500	1.13	.839	Plasmalogen
pyridoxate	1.34	.0220	1.21	.902	Vitamin B6 Metabolism
1-(1-enyl-stearoyl)-2-arachidonoyl-GPE (P-18:0/20:4)*	1.33	.0110	1.10	.828	Plasmalogen
1-(1-enyl-palmitoyl)-2-oleoyl-GPE (P-16:0/18:1)*	1.32	.0270	1.18	.895	Plasmalogen
sphingomyelin (d17:1/14:0, d16:1/15:0)*	1.32	.0250	1.02	.769	Sphingomyelins
1,2-dipalmitoyl-GPC (16:0/16:0)	1.31	.0210	1.18	.901	Phosphatidylcholine (PC)
erythritol	1.31	.00800	1.22	.927	Food Component/Plant
histidine betaine (hercynine)*	1.31	.0440	1.09	.830	Food Component/Plant
1-palmitoyl-2-oleoyl-GPC (16:0/18:1)	1.30	.0180	1.08	.834	Phosphatidylcholine (PC)
2-(4-hydroxyphenyl)propionate	1.30	.0100	1.16	.895	Benzoate Metabolism
1-palmitoyl-GPC (16:0)	1.29	.000	1.12	.868	Lysophospholipid
4-cholesten-3-one	1.29	.0130	1.12	.870	Sterol
taurocholenate sulfate*	1.29	.0450	1.08	.838	Secondary Bile Acid Metabolism
stachydrine	1.29	.0110	1.15	.893	Food Component/Plant
hypotaurine	1.28	.00900	1.14	.887	Methionine, Cysteine, SAM and Taurine Metabolism
7-alpha-hydroxy-3-oxo-4-cholestenoate (7-Hoca)	1.27	.0480	1.20	.944	Sterol
methylphosphate	1.27	.0180	1.08	.848	Purine and Pyrimidine Metabolism
3-hydroxy-2-ethylpropionate	1.25	.0130	1.10	.879	Leucine, Isoleucine and Valine Metabolism
**deoxycholic acid 12-sulfate***	1.25	.0390	1.06	.851	Secondary Bile Acid Metabolism
pantothenate	1.25	.0130	1.07	.856	Pantothenate and CoA Metabolism
N-acetyl-3-methylhistidine*	1.24	.00600	1.09	.878	Histidine Metabolism
1-(1-enyl-palmitoyl)-2-palmitoyl-GPC (P-16:0/16:0)*	1.24	.0140	1.14	.919	Plasmalogen
mevalonate	1.24	.0450	1.15	.925	Mevalonate Metabolism
gamma-glutamylglycine	1.23	.0200	1.17	.950	Gamma-glutamyl Amino Acid

cholesterol sulfate	**1.23**	.0340	1.08	.875	Sterol
isocitric lactone	**1.22**	.0460	1.08	.884	TCA Cycle
1-stearoyl-2-oleoyl-GPC (18:0/18:1)	**1.20**	.0440	1.07	.892	Phosphatidylcholine (PC)
S-methylglutathione	**1.19**	.0360	1.11	.931	Glutathione Metabolism
homostachydrine*	**1.19**	.0380	1.09	.915	Food Component/Plant
octadecanedioylcarnitine (C18-DC)*	**1.16**	.0460	1.00	.860	Fatty Acid Metabolism (Acyl Carnitine, Dicarboxylate)
2-O-methylascorbic acid	**1.16**	.0280	1.08	.927	Ascorbate and Aldarate Metabolism
(N(1) + N(8))-acetylspermidine	**1.15**	.0210	1.07	.931	Polyamine Metabolism
1-palmitoyl-2-stearoyl-GPC (16:0/18:0)	**1.14**	.0110	1.09	.960	Phosphatidylcholine (PC)
sphingomyelin (d18:1/14:0, d16:1/16:0)*	**1.14**	.0430	1.06	.930	Sphingomyelins
sphingomyelin (d18:2/16:0, d18:1/16:1)*	**1.13**	.0210	1.06	.940	Sphingomyelins
pipecolate	**1.12**	.0330	1.05	.938	Lysine Metabolism
adenosine 5'-monophosphate (AMP)	**1.12**	.0420	1.04	.927	Purine Metabolism, Adenine containing
glycine	**1.12**	.0430	1.06	.944	Glycine, Serine and Threonine Metabolism

Table S7. Differentially detected endogenous metabolites in DRG samples of untreated wild-type mice or OATP1B2-deficient (KO) mice. Names in red indicate previously reported putative OATP1B-type transport biomarkers (18). Negative fold change indicates lower levels in DRG of OATP1B2-deficient mice. Statistical analysis was performed using Welch's two-sample *t*-test.

Biochemical Name	Fold of Change KO/WT	*P*-value KO/WT	Mean value KO	Mean value WT	Sub Pathway
N6-methyllysine	0.500	.0420	.516	1.02	Lysine Metabolism
nervonoylcarnitine (C24:1)*	0.590	.0180	.694	1.18	Fatty Acid Metabolism (Acyl Carnitine, Monounsaturated)
lignoceroyl sphingomyelin (d18:1/24:0)	0.590	.0360	.779	1.32	Sphingomyelins
cys-gly, oxidized	0.620	.0310	.672	1.09	Glutathione Metabolism
α-tocopherol	0.730	.0310	.853	1.16	Tocopherol Metabolism
**chenodeoxycholic acid 24-glucuronide**	**NA**	NA	NA	NA	Primary Bile Acid Metabolism
**alpha-muricholate**	**NA**	NA	NA	NA	Primary Bile Acid Metabolism
**ursocholate**	**NA**	NA	NA	NA	Secondary Bile Acid Metabolism
**beta-muricholate**	**NA**	NA	NA	NA	Primary Bile Acid Metabolism
**hyodeoxycholate**	**NA**	NA	NA	NA	Secondary Bile Acid Metabolism
**cholate**	**27.8**	.000	1.24	.0447	Primary Bile Acid Metabolism
**deoxycholate**	**9.41**	.000	3.39	.361	Secondary Bile Acid Metabolism
**bilirubin (E,E)***	**NA**	NA	NA	NA	Hemoglobin and Porphyrin Metabolism
**hyocholate**	**NA**	NA	NA	NA	Secondary Bile Acid Metabolism
**bilirubin (Z,Z)**	**NA**	NA	NA	NA	Hemoglobin and Porphyrin Metabolism
**tetradecanedioate (C14-DC)**	**NA**	NA	NA	NA	Fatty Acid, Dicarboxylate
**ursodeoxycholate**	**NA**	NA	NA	NA	Secondary Bile Acid Metabolism
**chenodeoxycholate**	**NA**	NA	NA	NA	Primary Bile Acid Metabolism
**taurodeoxycholate**	**NA**	NA	NA	NA	Secondary Bile Acid Metabolism
**hexadecanedioate (C16-DC)**	**NA**	NA	NA	NA	Fatty Acid, Dicarboxylate
**taurodeoxycholic acid 3-sulfate**	**NA**	NA	NA	NA	Secondary Bile Acid Metabolism
**octadecanedioate (C18-DC)**	**NA**	NA	NA	NA	Fatty Acid, Dicarboxylate
**deoxycholic acid 12-sulfate***	**NA**	NA	NA	NA	Secondary Bile Acid Metabolism

References

1. Lamagna C, Scapini P, van Ziffle JA, DeFranco AL, and Lowell CA. Hyperactivated MyD88 signaling in dendritic cells, through specific deletion of Lyn kinase, causes severe autoimmunity and inflammation. *Proc Natl Acad Sci U S A* 2013;110(35):E3311-E20.
2. Huang KM, Leblanc AF, Uddin ME, Kim JY, Chen M, Eisenmann ED, et al. Neuronal uptake transporters contribute to oxaliplatin neurotoxicity in mice. *J Clin Invest.* 2020;130(9):4601-6.
3. Leblanc AF, Sprowl JA, Alberti P, Chiorazzi A, Arnold WD, Gibson AA, et al. OATP1B2 deficiency protects against paclitaxel-induced neurotoxicity. *Journal of Clinical Investigation.* 2018;128(2):816-25.
4. Chiorazzi A, Wozniak KM, Rais R, Wu Y, Gadiano AJ, Farah MH, et al. Ghrelin agonist HM01 attenuates chemotherapy-induced neurotoxicity in rodent models. *Eur J Pharmacol.* 2018;840:89-103.
5. Aten S, Page CE, Kalidindi A, Wheaton K, Niraula A, Godbout JP, et al. miR-132/212 is induced by stress and its dysregulation triggers anxiety-related behavior. *Neuropharmacology.* 2019;144:256-70.
6. Shi X, Bai H, Wang J, Wang J, Huang L, He M, et al. Behavioral Assessment of Sensory, Motor, Emotion, and Cognition in Rodent Models of Intracerebral Hemorrhage. *Front Neurol.* 2021;12:667511.
7. Basso DM, Fisher LC, Anderson AJ, Jakeman LB, McTigue DM, and Popovich PG. Basso Mouse Scale for locomotion detects differences in recovery after spinal cord injury in five common mouse strains. *J Neurotrauma.* 2006;23(5):635-59.
8. Leblanc AF, Huang KM, Uddin ME, Anderson JT, Chen M, and Hu S. Murine pharmacokinetic studies. *Bio-protocol.* 2018;8(20).
9. Tobias F, McIntosh JC, LaBonia GJ, Boyce MW, Lockett MR, and Hummon AB. Developing a drug screening platform: MALDI-mass spectrometry imaging of paper-based cultures. *Anal Chem.* 2019;91(24):15370-6.
10. Wozniak KM, Vornov JJ, Wu Y, Liu Y, Carozzi VA, Rodriguez-Menendez V, et al. Peripheral neuropathy induced by microtubule-targeted chemotherapies: insights into acute injury and long-term recovery. *Cancer Res.* 2018;78(3):817-29.
11. de Graan A-JM, Lancaster CS, Obaidat A, Hagenbuch B, Elens L, Friberg LE, et al. Influence of polymorphic OATP1B-type carriers on the disposition of docetaxel. *Clin Cancer Res.* 2012;18(16):4433-40.
12. Hagenbuch B, and Stieger B. The SLCO (former SLC21) superfamily of transporters. *Mol Aspects Med.* 2013;34(2-3):396-412.
13. Uddin ME, Talebi Z, Chen S, Jin Y, Gibson AA, Noonan AM, et al. In Vitro and In Vivo Inhibition of MATE1 by Tyrosine Kinase Inhibitors. *Pharmaceutics.* 2021;13(12).
14. Glaeser H, Bailey D, Dresser G, Gregor J, Schwarz U, McGrath J, et al. Intestinal drug transporter expression and the impact of grapefruit juice in humans. *Clin Pharmacol Ther.* 2007;81(3):362-70.
15. McLeod C, Gout AM, Zhou X, Thrasher A, Rahbarinia D, Brady SW, et al. St. Jude cloud: A pediatric cancer genomic data-sharing ecosystem. *Cancer Discov.* 2021;11(5):1082-99.
16. Yee SW, Gong L, Badagnani I, Giacomini KM, Klein TE, and Altman RB. SLC19A1 pharmacogenomics summary. *Pharmacogenet Genomics.* 2010;20(11):708.
17. Diouf B, Crews KR, Lew G, Pei D, Cheng C, Bao J, et al. Association of an inherited genetic variant with vincristine-related peripheral neuropathy in children with acute lymphoblastic leukemia. *Jama.* 2015;313(8):815-23.

18. Li Y, Talebi Z, Chen X, Sparreboom A, and Hu S. Endogenous biomarkers for SLC transporter-mediated drug-drug interaction evaluation. *Molecules.* 2021;26(18):5500.
